# Water Released From Vessel Embolism May Temporarily Halt Further Embolism in Tropical Dipterocarp Trees

**DOI:** 10.1111/pce.70647

**Published:** 2026-07-02

**Authors:** Arne Scheire, Paulo Roberto de Lima Bittencourt, Palasiah Jotan, Jehová Lourenço, Radim Matula, Martin Svátek, Reuben Nilus, Mohd. Aminur Faiz Bin Suis, Rolando Robert, Steven Jansen, Maurizio Mencuccini, Karina Williams, Tommaso Jucker, Lindsay F. Banin, David F. R. P. Burslem, Lucy Rowland

**Affiliations:** ^1^ Department of Geography Faculty of Environment, Science and Economy, University of Exeter Exeter UK; ^2^ South East Asia Rainforest Research Partnership (SEARRP) Kota Kinabalu Malaysia; ^3^ Department of Forest Ecology Faculty of Forestry and Wood Sciences, Czech University of Life Sciences Prague Prague Czech Republic; ^4^ Royal Botanic Gardens, Kew Richmond UK; ^5^ Department of Forest Botany Dendrology and Geobiocoenology, Faculty of Forestry and Wood Technology, Mendel University Brno Czech Republic; ^6^ Sabah Forestry Department Forest Research Centre Sandakan Malaysia; ^7^ Research Management Centre, Universiti Malaysia Sabah Kota Kinabalu Malaysia; ^8^ Institute of Botany, Ulm University Ulm Germany; ^9^ CREAF, Cerdanyola del Vallès Barcelona Spain; ^10^ ICREA Barcelona Spain; ^11^ Met Office Exeter UK; ^12^ School of Biological Sciences, University of Bristol Bristol UK; ^13^ UK Centre for Ecology & Hydrology Penicuik UK; ^14^ School of Biological Sciences, University of Aberdeen Aberdeen UK

**Keywords:** dipterocarps, drought resilience, embolism, tropical rainforest, water potential

## Abstract

The formation of air bubbles in the xylem, known as embolism, reduces hydraulic conductivity and can ultimately lead to catastrophic failure of a tree's water transport system. When a vessel embolises, it releases water into the surrounding xylem tissue. Yet, how this release affects plant water status and further embolism spread remains poorly studied, especially in tropical trees. We examined xylem vulnerability curves from branches of 38 trees representing five dipterocarp species growing in a Bornean tropical rainforest. While branch water potential progressively declined as branches desiccated over time, temporary increases in branch water potential were repeatedly observed across all trees. These increases predominantly took place in the water potential range where embolism occurred, indicating that the observed increases most likely resulted from water released from embolised vessels. Our findings suggest that water release from embolism may temporarily buffer water potential declines and further embolism spread during desiccation in dipterocarp trees, although the extent to which this buffering capacity is present in other tropical and temperate species should be investigated further. Our study provides novel insights into xylem water dynamics and the mechanisms by which trees may sustain hydraulic function during periods of water stress, potentially enhancing their drought resilience.

## Summary statement

Water released from embolised vessels in five dipterocarp species temporarily increased water potential of excised branches during desiccation, demonstrating a novel mechanism by which tropical trees may resist catastrophic embolism and enhance their resilience to drought.

## 1 Introduction

Understanding how trees cope with periods of water stress is crucial to predicting how tropical forests will respond to increased drought frequency and intensity in the future (Trenberth et al. 2014; Choat et al. 2018; McDowell et al. 2018; IPCC 2022). This is particularly important for Southeast Asian tropical forests, which store large amounts of carbon (Qie et al. 2017; Estoque et al. 2019) but remain understudied relative to Amazonian forests (Hubau et al. 2020; Gatti et al. 2021). One approach to determine the vulnerability of trees to drought is by measuring their ability to resist xylem embolism, which occurs when decreasing xylem water potential leads to the formation of air bubbles in the vessels, reducing hydraulic conductivity (Sperry and Tyree 1988; Choat et al. 2012; Tyree and Zimmermann 2013). The water potential corresponding to 50% loss of conductivity (*P*_50_) has been widely used for comparisons of xylem vulnerability to drought across species and correlates with the risk of plant mortality to drought (McDowell et al. 2008; Blackman et al. 2009; Brodribb and Cochard 2009; Meinzer et al. 2009; Brodribb et al. 2010; Choat et al. 2012; Urli et al. 2013; Rowland et al. 2015; Choat et al. 2018).

To prevent hydraulic failure, trees must avoid embolism (Tyree and Sperry 1988; Urli et al. 2013; Gleason et al. 2014). However, during intense or prolonged periods of water stress, this is often not possible and low water potentials cause embolism in tropical trees (Sobrado 1997; Meinzer et al. 2008; Johnson et al. 2012). The formation of embolism is a complex process influenced by multiple factors beyond just xylem water potential. While low water potential is a key driver, embolism also depends on the proximity to gas under atmospheric pressure, interconduit connectivity, the degree of hydraulic compartmentalisation, vessel dimensions, and the rates of axial and radial gas diffusion (Wang, Liu, et al. 2015; Wang, Pan, et al. 2015; Silva et al. 2024). Additionally, embolised conduits themselves can accelerate further embolism spread in neighbouring conduits, potentially leading to catastrophic hydraulic failure if a considerable fraction of the water‐conducting capacity is lost (Tyree and Sperry 1989). While this process is generally assumed to be irreversible without a resupply of water, it is possible that trees can slow down this process and delay further embolism by redistributing internally stored water supplies, potentially prolonging survival and preventing mortality of the entire plant (Hölttä et al. 2009; Meinzer et al. 2009). Despite its importance for predicting tropical forest responses to water stress, the timing of hydraulic failure in trees and the complex mechanisms that may limit or delay it remain poorly studied in tropical trees (McDowell et al. 2018; Powers et al. 2020; Bittencourt et al. 2022).

During persistent water shortage, tree water status progressively declines and various plant tissues sequentially release water into the transpiration stream (Wei et al. 2023). This process typically occurs in three phases: an initial phase with rapid water release from intercellular and capillary spaces, such as the apoplastic lumina of fibres; a second phase where symplastic water is discharged from intracellular storage compartments such as axial or ray parenchyma cells; and a third phase where water is released from conduits (i.e., vessels or tracheids) due to embolism formation (Tyree and Yang 1990; Tyree and Zimmermann 2002; Gleason et al. 2014; Jupa et al. 2016; Wei et al. 2023). The release of this apoplastic and symplastic water relative to a given change in water potential is termed capacitance, a process which, although poorly studied, plays a crucial role in maintaining tree hydraulic integrity (Salomón et al. 2017) and has the potential to significantly buffer against short‐term drought stress (Tyree and Yang 1990; Hölttä et al. 2009; Scholz et al. 2011; Vergeynst et al. 2015). In addition, the residual conductance from leaf and bark—representing water loss that persists even when stomata are closed—also plays a critical role in controlling plant water loss, the development of xylem embolism and survival time during drought (Duursma et al. 2019; Burlett et al. 2025).

When a conduit embolises, it becomes filled with water vapour or gas, and nearly all the liquid water inside it is released to the surrounding xylem tissue (Hölttä et al. 2009; Gleason et al. 2014; Wang, Liu, et al. 2015; Wang, Pan, et al. 2015; Silva et al. 2024). Unlike other sources of water release from plant tissues during desiccation, the water released by embolised conduits is unbound and possesses high potential energy, which can temporarily increase the plant's water potential (Zimmermann 1983). It is important to note, however, that this water release results from a phase change during embolism formation and is inherently hysteretic; as such, it is not reversible in the manner of capillary or elastic water storage and should not be interpreted as true capacitance. These temporary increases in water potential are likely to scale with the amount of embolism and conduit dimensions (Hölttä et al. 2009; Wang, Liu, et al. 2015; Wang, Pan, et al. 2015). If these increases are large enough to offset the ongoing decline in water potential due to evaporative water loss during periods of water stress, they may contribute towards preventing further loss of conductivity and halting, at least temporarily, further embolism propagation to neighbouring conduits (Hölttä et al. 2009). It has also been suggested that local extraction of gas in a recently embolised conduit may provide a temporary buffer to embolism spread in a neighbouring conduit (Silva et al. 2024). Thus, the capacitative release of water from embolised conduits may be one of few potential mechanisms that could allow trees to reduce the risk of further embolism spread without requiring an increase in plant water volume. As a result, depending on the magnitude and duration of this buffering effect from capacitance, a tree may experience reduced overall damage to the hydraulic system. This would reduce the costs of repairing or replacing embolised xylem, potentially limiting the impact of drought events on woody plants (Scholz et al. 2011). However, there are limited ways to directly measure tree capacitance (Scholz et al. 2011) and indirect measures remain complex to implement due to the heterogeneous nature of wood, especially at the whole‐tree scale (Meinzer et al. 2004; 2008; Scholz et al. 2011; Pfautsch et al. 2015; Vergeynst et al. 2015; Jupa et al. 2016; Salomón et al. 2017; Santiago et al. 2018; Siddiq et al. 2019; Wei et al. 2023). Consequently, quantitative estimates of tree capacitance are often lacking in studies on drought resistance (Vergeynst et al. 2015) and the role of capacitance in slowing or temporarily reversing the effects of embolism remains poorly understood (Wei et al. 2023).

Most evaluations of xylem vulnerability to embolism focus on branch xylem (Gleason et al. 2014; Vergeynst et al. 2015; Oliveira et al. 2019; Trifilò et al. 2019; Bittencourt et al. 2022; Tavares et al. 2023; Wei et al. 2023), with relatively fewer studies exploring vulnerability in leaves (Blackman et al. 2014; Brodribb, Bienaimé, et al. 2016; Brodribb, Skelton, et al. 2016; Scoffoni et al. 2017a; 2017b; Lucani 2019; Ziegler et al. 2019; Creek et al. 2020; Powers et al. 2020; Tan et al. 2020; Yao et al. 2021; 2023). The leaf vascular system plays a crucial role in supplying the mesophyll with sufficient water for photosynthesis, governing the rate of water and CO_2_ exchange between plants and the atmosphere (Brodribb and Holbrook 2006). Previous studies have shown that embolism‐induced declines in leaf hydraulic conductivity can strongly impact the growth, productivity and overall water balance of plants (Sack and Holbrook 2006; McDowell et al. 2008; Anderegg et al. 2015). Given the crucial role of leaves in the whole‐plant hydraulic system, understanding which mechanisms can help prevent hydraulic failure in the leaves is essential, as these may strongly dictate plant responses to water stress, drought‐induced mortality and drought adaptive capacity (Brodribb and Holbrook 2006; Blackman et al. 2010; Brodribb, Bienaimé, et al. 2016; Brodribb, Skelton, et al. 2016).

As trees grow larger, their water storage capacity and the capacitive effect of embolism are expected to increase (Phillips et al. 2003; Hölttä et al. 2009; Scholz et al. 2011), which could be particularly important for alleviating drought in tall trees (Scholz et al. 2011), such as those found in Southeast Asia (Banin et al. 2012; 2014). However, quantifying the amount and physiological impact of water released from embolised vessels remains challenging. While the amount of water released by vessel embolism can be estimated using vessel anatomy and branch structural traits, it is still unclear how this relates to fluctuations in water potential of the surrounding tissues and how much of this water eventually ends up in the leaves (Wei et al. 2023). A modelling study by Hölttä et al. (2009) demonstrated that water released from embolism could protect a plant from further embolism over short intervals. However, empirical studies reporting increases in water potential are extremely scarce (i.e., no reported increases in Lamarque et al. 2018 and Avilla et al. 2022; but see Lo Gullo and Salleo 1992). Consequently, quantitative estimates of the timing and magnitude of this buffering effect are lacking. Given that plants often encounter significant embolism only under extreme hydraulic stress (Meinzer et al. 2009; Urli et al. 2013; Guan et al. 2022), understanding if and where along the gradual dehydration phases of plants these water potential increases occur is crucial.

In this study, we utilise high temporal resolution measurements obtained with the optical method and psychrometers to investigate embolism formation in leaves and the dynamic changes in branch water potential during desiccation, and explore the potential impact on tree hydraulic functioning in Southeast Asia's tallest tropical forests. We focus on five species belonging to the family of Dipterocarpaceae, containing the tallest tropical trees in the world, and explore the following questions:

1. Can increases in branch water potential be detected during desiccation?
2. Do increases in water potential coincide with periods of maximum rates of embolism spread?
3. Is the water released from vessel embolism likely to be physiologically relevant?

## 2 Methods

### 2.1 Study Site and Sample Collection

This study took place in a mixed dipterocarp forest in the Kabili‐Sepilok Forest Reserve in Sabah, Malaysian Borneo (lat. 5°52′ 34" N, long. 117°56′ 58" E) from April‐June in 2022. The region has a mean (± SD) monthly temperature of 26.5 ± 0.6°C and mean annual precipitation of 2640 ± 675 mm, with no dry season (< 100 mm monthly rainfall) except under extreme drought conditions that occasionally happen during El Niño periods (Bittencourt et al. 2022). Here, plant material was collected in three permanent alluvial forest plots of 4 ha, characterised by nutrient rich and clayey soils (Nilus et al. 2004; 2011; Bittencourt et al. 2022) where dipterocarp trees taller than 70 m can occur (Jucker et al. 2018). The distance between these plots was less than 1 km, so climatic differences across them should be small. Within the plots, five dipterocarp species were selected from multiple genera to represent a broad phylogenetic spectrum, enabling more robust generalisations for the Dipterocarpaceae family. These genera (*Dipterocarpus*, *Dryobalanops*, *Parashorea* and *Rubroshorea*) are widespread across the Asian tropics (WFO 2023) and are also the most abundant in Borneo (Fox 1972; Romell 2011). The species selected were *Dipterocarpus caudiferus*, *Dryobalanops lanceolata*, *Parashorea malaanonan*, *Parashorea tomentella* and *Rubroshorea johorensis* (WFO 2023), and encompassed a wide range of ecological strategies, from slow‐growing, extremely shade‐tolerant trees to light‐demanding fast growers (Scheire 2025; Jotan et al. in revision).

In total, 38 trees were sampled, ranging in height from 7 to 71 m tall and diameter at breast height (1.3 m) from 9 to 153 cm. For each species, a minimum of six trees was sampled. The aim of the sampling design was to maximise the height variation across trees (i.e., to obtain a distribution of tree heights ranging from the shortest to tallest individuals of that species; Figure S1). Tree crowns were accessed by experienced tree climbers using double rope climbing techniques. Total tree height and branch sampling height were measured using a laser range finder (*Forestry Pro II; Nikon, Tokyo, Japan*), and height values were confirmed by the tree climbers. For each tree, one branch of approximately 1–2 m long was collected from the top of the crown (i.e., from the uppermost branch). Depending on individual tree crown size, additional branches were also sampled from the lowest branch of the crown and from an intermediary branch. Overall, 15 trees had top, middle and bottom branches sampled, 10 had top and bottom branches sampled, and 13 of the smaller trees only had top branches sampled due to their small crown depths. Upon excision, branch samples were quickly brought down to the ground and placed inside black plastic bags containing moist tissue paper to avoid dehydration while transporting the samples back to the lab at the Forest Research Centre, 10–30 min’ walk from the plots. When branch samples arrived at the lab, 1–2 h after being cut, they were placed inside buckets filled with water and recut under water. Branch samples were then stored in plastic bags with the cut end of the branch remaining submerged in water and were rehydrated overnight to ensure that each sample had the same starting condition. Leaf measurements were conducted using the branch as the sampling unit, meaning that all leaves from an individual branch were considered equivalent.

### 2.2 Constructing High‐Resolution Leaf Vulnerability Curves

Leaf vulnerability to embolism was measured using the optical method (Brodribb, Bienaimé, et al. 2016; Brodribb, Skelton, et al. 2016). Conventional methods for constructing xylem vulnerability curves implement a sigmoidal or Weibull function to fit conductivity loss to water potential decline (Pammenter and Van der Willigen 1998; Ogle et al. 2009; Duursma and Choat 2017). These fits typically rely on relatively few water potential measurements, spread over significant time periods (hours to days) and use the fitting procedure to interpolate the remaining water potential between full rehydration and complete hydraulic failure (Duursma and Choat 2017). This means that the curves may not have sufficient resolution to detect releases of stored water and their impact on the water potential of the branch segments. The combination of the optical method (Brodribb, Bienaimé, et al. 2016; Brodribb, Skelton, et al. 2016; Lucani 2019), in which a scanner or microscope is used to visually detect embolism formation within the xylem, with automated xylem water potential measurements (e.g., using psychrometers), offers a finer time‐resolution to detect water potential increases. Because this approach measures water potential continuously, at much finer intervals (e.g., every 10 min; Brodribb et al. 2017), it enables a more precise investigation of water potential and embolism fluctuations over time and therefore offers a greater capacity to capture water potential increases.

The optical method works by capturing images of the leaf xylem at regular intervals over a period of time using transmitted light and analysing the differences in light intensity that occur when vessels in the xylem embolise (Brodribb, Bienaimé, et al. 2016; Brodribb, Skelton, et al. 2016; see Lucani 2019 for details of image processing). For each fully rehydrated branch, one leaf was scanned every 10 min as it was left to dry out while still being attached to the branch, using *Epson Perfection V600 Photo* scanners at 4200 DPI resolution. Simultaneously, branch water potential (ψ_branch_) was monitored every 10 min using a stem psychrometer (*PSY1, ICT International, Armidale, NSW, Australia*). Given that the whole branch sample was disconnected from the soil and that stomata were closed during the experiment, the water potential of the entire branch sample was assumed to be close to equilibrium (Brodribb and Holbrook 2003; Lucani 2019). Leaves were scanned for at least 36 h, and were scanned for longer if ψ_branch_ continued to decline or leaves had not turned brown, at which point leaf xylem vessels were assumed to be fully embolised. Subsequently, xylem embolism per image was quantified in ImageJ (v1.54 h; Schneider et al. 2012) as the count of embolised pixels per image divided by the total cumulative count of embolised pixels measured across the entire experiment to represent the percentage of total cumulative embolism (Lucani 2019; Creek et al. 2020). The percentage of embolism at the time of image capture was then linked to ψ_branch_ from the psychrometer. Leaf xylem vulnerability curves were then constructed (relationship of percentage cumulative embolism plotted against ψ_branch_) to extract *P*_50_, the ψ_branch_ at which 50% of total cumulative leaf embolism had occurred. Leaf *P*_12_ and *P*_88_ were also calculated. These leaf vulnerability experiments were conducted as part of a broader field campaign in which woody vulnerability curves were also measured using the pneumatic method (Pereira et al. 2020; Bittencourt et al. 2026). As a result, the optical method was applied only to leaves, meaning that comparable high‐resolution vulnerability curves were not available for the wood. Despite this high‐resolution of the optical method, we note that all methods involved in measuring leaf and branch vulnerability curves make quantifying the direct impact of capacitance on embolism formation complex (Cochard et al. 2013). This is because they are generally carried out on relatively small branch segments, which may limit the amount of water available to be released (Hölttä et al. 2009). Furthermore, these branches have also been excised from the tree, which accelerates experimental desiccation relative to natural drought conditions and potentially masks the impacts of increases in water potential.

### 2.3 Psychrometer Measurement Accuracy

Given the focus of our study on fluctuations in ψ_branch_ during branch desiccation, measurement accuracy was crucial. While psychrometers provide continuous ψ_branch_ measurements, the precision of readings below 0.1 MPa remains uncertain, as they may be influenced by specific parameter settings, such as cooling and waiting times. To minimise potential artifacts, our psychrometer settings followed best practices and recommendations from (published) studies (Brodribb et al. 2017; Lucani 2019) and the psychrometer manufacturer (ICT International 2014; Dixon and Downey 2017). In one of these studies, cooling time during psychrometric measurements was increased throughout the experiment (i.e., from 5 to 30 s in Brodribb et al. 2017) as ψ_branch_ became more negative to ensure sufficient condensation on the thermocouple and a stable wet‐bulb depression. In practice, however, guidance on how to adjust cooling time during progressive desiccation is limited, particularly for understudied tropical species, and arbitrary changes without clear benchmarks could introduce additional uncertainty. For these reasons, cooling time for our studied species was held constant across the entire experiment, following the manufacturer's default settings (10 s cooling, 6 s waiting, 15 s pre‐measurement delay; ICT International 2014; Dixon and Downey 2017). We acknowledge that holding cooling time constant may introduce a systematic bias at more negative ψ_branch_. However, all experiments were conducted with identical psychrometer settings, ensuring that any potential bias was consistent across samples and thus not a confounding factor when interpreting the results. In addition, we collected preliminary ψ_branch_ measurements over several hours on desiccating branches of our study species to evaluate the performance of the chosen psychrometer settings. These tests indicated that the chosen parameters produced stable readings, with no evidence of irregularities such as premature plateau loss (which would indicate false readings) or failed measurements. We are therefore confident that we applied the most appropriate protocol available to ensure measurement accuracy and minimise bias.

### 2.4 Evaluating Changes in Water Potential

Changes in ψ_branch_ over time were initially calculated as the difference between consecutive psychrometer measurements (i.e., delta water potential every 10 min; Δψ_branch_). To more accurately isolate ψ_branch_ increases associated with embolism‐related water release, the time series for each branch was detrended to remove the overall secular decline during dehydration. This was achieved by fitting a locally weighted regression (LOESS) to the ψ_branch_ measurements as a function of time, using a smoothing span of 0.3. The span was empirically determined by fitting a series of LOESS models for each branch and selecting the mean span across branches that minimised spurious positive excursions in the detrended water potential signal. The fitted LOESS curve represents the underlying decline in ψ_branch_. The detrended signal was obtained by subtracting this curve from the original ψ_branch_ measurements, isolating transient positive and negative excursions. Negative excursions were assumed to reflect background noise and were used to estimate its mean magnitude. To isolate positive excursions associated with embolism‐related water release, the mean magnitude of negative excursions was subtracted from the positive excursions. Any positive excursions smaller than the estimated background noise were set to zero and the magnitude of the remaining positive excursions was calculated as the difference between the fitted ψ_branch_ post‐detrending and the actual psychrometer measurement.

During the experiments, room temperature and relative humidity were simultaneously measured every 5 min using *Sensirion SHTC3* temperature and air humidity sensors coupled to a custom datalogger. The temperature in the lab during measurements was 20.2 ± 0.50°C on average (mean ± SD) and ranged from 19.0°C to 22.0°C. The relative humidity averaged 54.1 ± 6.0% (mean ± SD), with a range of 36.4%–76.0%. To evaluate the possibility that increases in ψ_branch_ (i.e., positive Δψ_branch_) may have been driven by changes in temperature and/or relative humidity, positive values for Δψ_branch_ were tested for correlation with the corresponding changes in room temperature and relative humidity. Increases in ψ_branch_ were not related to changes in temperature or relative humidity (Figures S2 and S3).

To evaluate the timing of increases in ψ_branch_ relative to hydraulic thresholds, we also obtained data on branch (i.e., woody) *P*_12_, *P*_50_ and *P*_88_, as well as the leaf turgor loss point (*TLP*) for each of the branches (for detailed methods see Bittencourt et al. 2026). To evaluate whether increases in ψ_branch_ scaled with the amount of ongoing embolism, the proximity of changes in ψ_branch_ to branch *P*_50_ (hereafter termed branch *P*_50_ difference) was determined by subtracting branch *P*_50_ from ψ_branch_ for each point in the respective vulnerability curve, with positive values indicating that the respective change in ψ_branch_ occurred before branch *P*_50_ (i.e., at a less negative water potential). This was also done for leaf *P*_50_ (leaf *P*_50_ difference). To evaluate similarity of embolism resistance between leaves and branches we compared their respective embolism ranges (i.e., *P*_12_–*P*_88_) and also tested for significant differences between leaf and branch *P*_50_ (see Table S1).

### 2.5 Physiological Relevance of Water Released From Woody Vessels

To evaluate the potential physiological relevance of water released from woody vessel embolism, we pursued three objectives: (1) estimate the amount of water released from woody vessels, (2) estimate the resulting increase in leaf water potential assuming all released water is distributed to the leaves and (3) compare the amount of water released from woody vessels with that from other sources of capacitance.

In order to estimate the water released from woody vessels, we required branch anatomical characteristics. We obtained these data at 1 m distance from the tip of the branch for each of the 75 branches, as described in Jotan et al. (in revision). Using a simple modelling approach, the water content inside the vessels of 1‐m‐long cylindrical branch segments was determined for 72 of our studied branches, for which wood anatomical features were available. Model parameters and equations are shown in Table S2. First, vessel lumen fraction was multiplied by branch xylem cross‐sectional area and branch segment length (1 m) to obtain the total xylem lumen volume, assuming that vessels were randomly distributed within the branch segment. Total vessel water content was then determined by converting xylem lumen volume (µm^3^) to the corresponding weight of water (grams). The water released from this theoretical branch segment at a given percentage of vessel embolism was then calculated as a fraction of the total vessel water content.

To investigate the potential impact of this water released on leaf function, we assumed all of this water would eventually be redistributed towards the leaves due to leaves being the primary sites of evaporation. The resulting increase in leaf water potential due to water released from vessel embolism was determined using leaf capacitance data. Area‐based leaf capacitance was measured at the leaf level using pressure‐volume curves, following Williams et al. (2017). For each branch, this measurement was then scaled up to total leaf capacitance, accounting for the total leaf area within 1 m of the branch tip, where the anatomical measurements were taken. Thus, total leaf capacitance was calculated as the amount of water (grams) leading to a 1 MPa increase for a given branch's leaf water potential. The mean (± 95% confidence interval) increase in leaf water potential was determined for all branches within and across species.

Importantly, our approach to estimate the amount of water released by embolism could be overestimated, as it is possible that not all of the water released ends up in the leaves, and some of it is lost through residual conductance through the bark. Consequently, the values derived from our analysis should be interpreted with caution and not considered empirical evidence of water redistribution from branch to leaf. Despite this uncertainty, we believe that our analysis is a useful exercise to investigate the potential physiological relevance of the water released from vessel embolism for leaf hydraulic function. Additionally, to put our estimates of the water released from vessels into perspective, for 66 sampled branches, we derived the ‘standardised water content’ (i.e., the water content per cm of branch length; g cm^−1^) from measurements of saturated water content in the sapwood and bark at 1 m distance from the tip of branches. These values were compared with our estimates of water content in the vessels, which are included in the sapwood. The relative contribution of bark, sapwood and vessels specifically to the standardised water content was calculated as a fraction of the standardised water content of bark and sapwood combined (termed ‘standardised water content fraction’). Next, by accounting for the respective tissue volumes per cm of branch length in each of our sampled branches, we calculated the amount of water likely to be released during severe desiccation (i.e., up to *P*_50_) per cm of branch length. This was done assuming that 20% of the water contained in the bark and non‐vessel sapwood (e.g., parenchyma and fibres; calculated as sapwood water content minus vessel water content) and 50% of the vessel water content is released at *P*_50_. A 20% release from bark and non‐vessel sapwood was used based on the branch desiccation experiments by Borchert and Pockman (2005) and Knipfer et al. (2019), as well as broader scientific literature indicating that living cells in these tissues are expected to stay above 80% relative water content to prevent structural damage. Thus, these calculations should provide realistic estimates of the total releasable water (g cm^−1^) within a physiologically relevant range.

### 2.6 Statistical Data Analysis

Analysis of variance was used to test whether the mean increase in ψ_branch_ significantly differed between species. Pearson's correlation analysis was used to test whether the increases in ψ_branch_ were significantly correlated with changes in room temperature and relative humidity. Wilcoxon signed‐rank test was used to test for significant differences between leaf and branch *P*_50_. Fixed effect linear models were used to test for significant relationships between the mean and cumulative increases in ψ_branch_ and branch height, with branch height as a fixed effect. All analyses were performed in the R statistical environment (v.4.2.2; R Core Team 2020). The ‘loess’ function from R's base package was used to fit the detrended ψ_branch_ time series. The ‘*aov*’ and ‘*cor. test*’ functions from R's base package were used for the analysis of variance and Pearson's correlation analysis, respectively. The ‘*lm*’ function from R's base package was used for fixed‐effect linear models.

## 3 Results

### 3.1 Can Increases in Branch Water Potential Be Detected During Desiccation?

All branches showed an overall progressive decline in water potential (ψ_branch_) during desiccation. However, temporary increases in ψ_branch_ were observed in the leaf xylem vulnerability curves of all 75 samples. For some curves, the increases in ψ_branch_ resulted in a clear ‘bump’ in the curve's general trajectory (Figure 1a,b), whereas for others this effect was less pronounced (Figure S4). Figure 1a,b illustrates the changes in ψ_branch_ over time for two example curves, with subpanels highlighting specific parts of the curves where increases in ψ_branch_ were greatest. Across branches, the mean increase in ψ_branch_ (i.e., positive Δψ_branch_ for each 10‐min interval; Figure 2a) was 0.036 ± 0.042 MPa (mean ± SD). The increases in ψ_branch_ were not related to changes in temperature or relative humidity (Figures S2 and S3). The maximum ψ_branch_ increase across branches ranged from 0.043 to 0.5552 MPa, averaging 0.214 ± 0.112 MPa (mean ± SD). The analysis of variance showed that the mean increase in ψ_branch_ did not differ significantly among species (*F*[4, 21,588] = 1.751, *p* = 0.136), which was 0.034 ± 0.033 MPa (mean ± SD) (*D. caudiferus*), 0.029 ± 0.033 MPa (*D. lanceolata*), 0.037 ± 0.044 MPa (*P. malaanonan*), 0.038 ± 0.041 MPa (*P. tomentella*) and 0.038 ± 0.049 MPa (*R. johorensis*).

**Figure 1:**
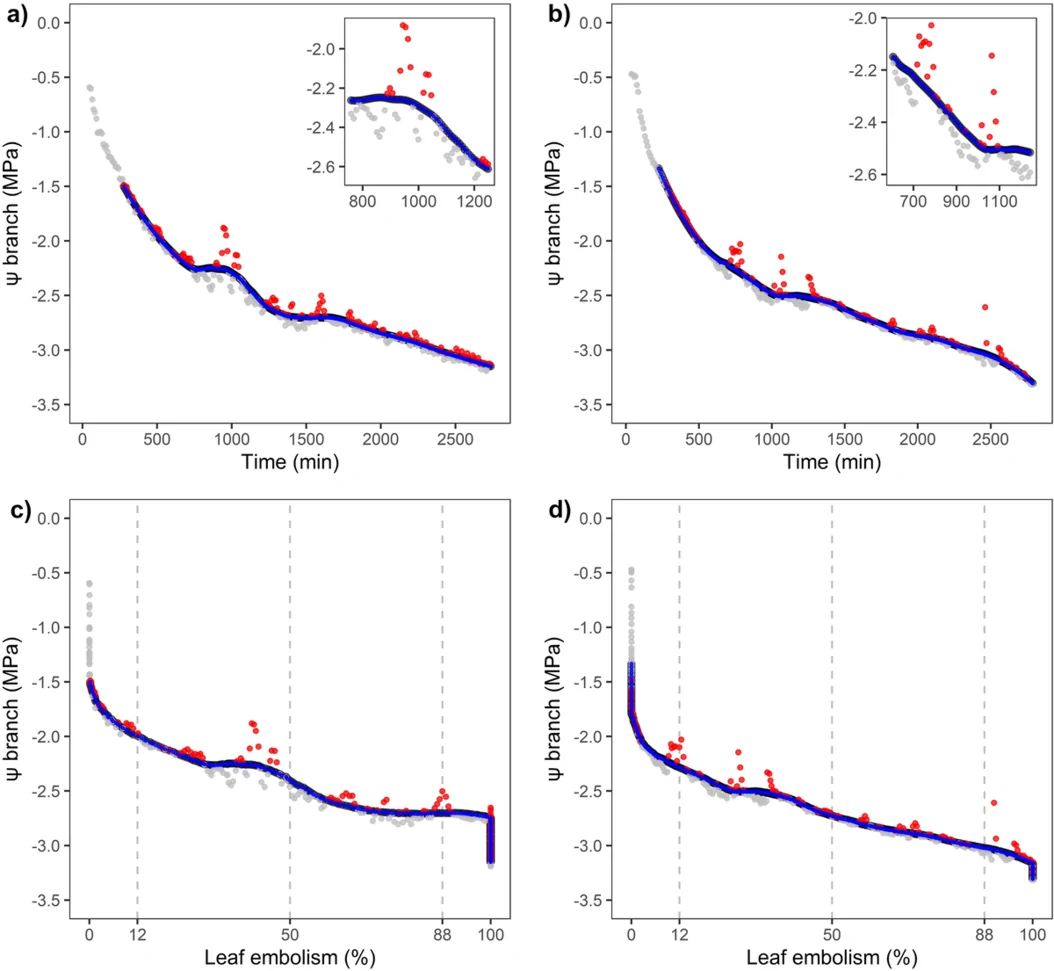
Changes in ψ_branch_ over time, measured every 10 min, for two samples of *Rubroshorea johorensis* (a, b). Subpanels in (a) and (b) highlight specific parts of the curves where increases in ψ_branch_ were largest. Grey and red points represent the original ψ_branch_ measured by the psychrometer. Points in time where ψ_branch_ increased (corrected for noise by detrending) are coloured in red. The blue line represents the fitted water potential after detrending. The corresponding leaf vulnerability curves (ψ_branch_ plotted against cumulative % leaf embolism) for both example curves in (a) and (b) are also shown (c, d) using the same colour legend. For additional examples, see Figure S4.

**Figure 2:**
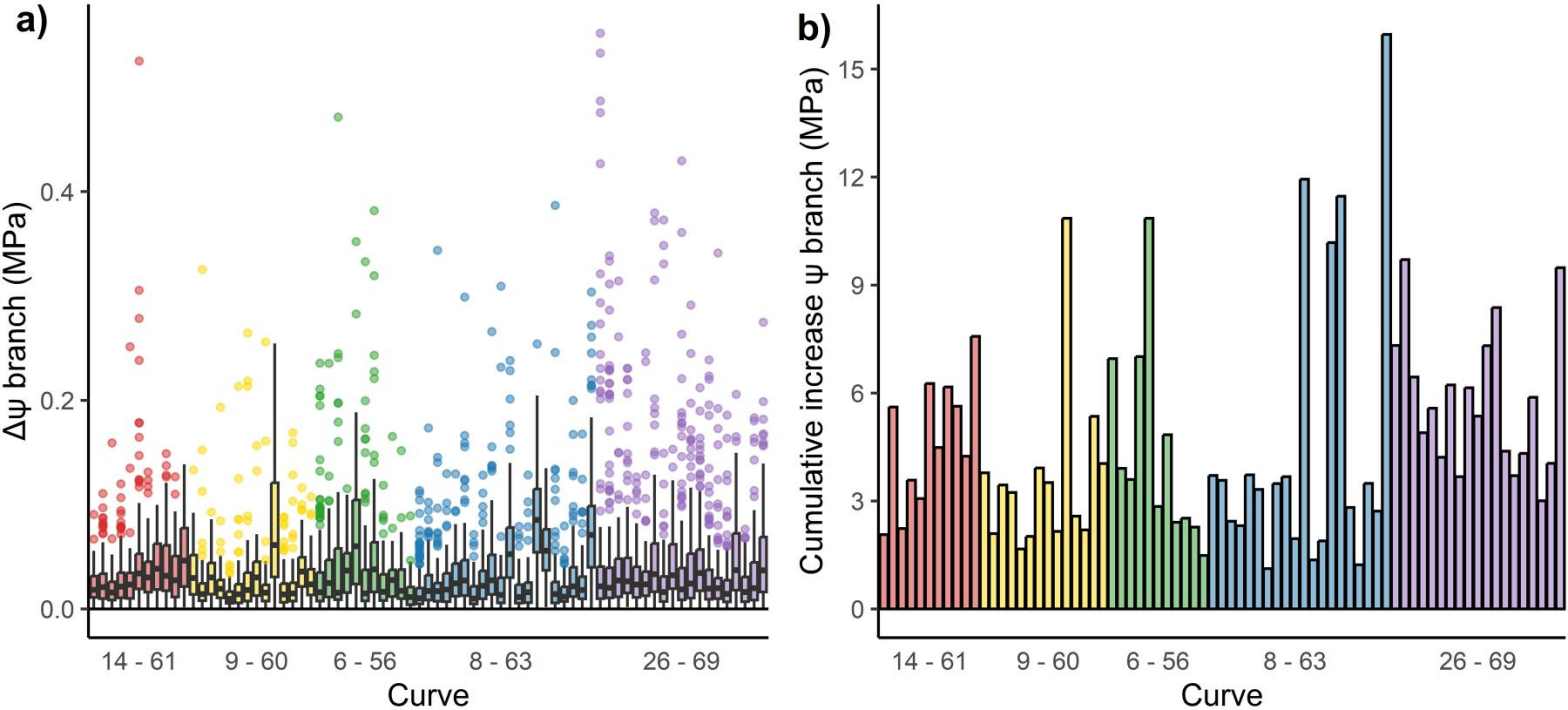
The boxplot in (a) shows the variability (i.e., median, interquartile range and whiskers) of increases in ψ_branch_ (i.e., positive Δψ_branch_) within each curve, for all curves. Each bar along the x‐axis represents one curve. Bars within species are in order of increasing branch sampling height (m). Red = *Dipterocarpus caudiferus*, yellow = *Dryobalanops lanceolata*, green = *Parashorea malaanonan*, blue = *Parashorea tomentella*, purple = *Rubroshorea johorensis*. Minimum and maximum branch sampling height (m) within species are displayed along the *x*‐axis. Individually plotted values represent outliers that were greater than 1.5 times the interquartile range above the third quartile. The barplot in (b) shows the total cumulative increased ψ_branch_ within each curve. [Color figure can be viewed at wileyonlinelibrary.com]

When the increases in ψ_branch_ within each curve were summed up, the total cumulative increased ψ_branch_ was 4.705 ± 2.900 MPa on average (mean ± SD), ranging from 1.120 to 15.962 MPa across branches (Figure 2b). The mean increase in ψ_branch_ and the total cumulative increased ψ_branch_ were significantly and positively related to branch sampling height for *D. caudiferus* only (*p* < 0.01 and *p* < 0.05, respectively; Table S3).

### 3.2 Do Increases in Water Potential Coincide With Periods of Maximum Rates of Embolism Spread?

The mean *P*_50_ values for leaf and branch xylem were closely aligned, and the leaf embolism range (*P*_12_–*P*_88_) fell entirely within the branch embolism range (Figure 3a). When all samples were pooled together, leaf and branch *P*_50_ differed significantly (*p* < 0.001), with branches reaching *P*_50_ on average (mean ± 95% CI) 0.26 ± 0.13 MPa before leaves (see Table S1). However, when examined within species, significant differences between leaf and branch *P*_50_ were observed only for *D. lanceolata* (*p* < 0.001), *P. tomentella* (*p* < 0.05) and *P. malaanonan* (*p* < 0.05), whereas no significant differences were detected for *R. johorensis* (*p* = 0.568) and *D. caudiferus* (*p* = 0.383). The frequency and magnitude of changes in ψ_branch_ varied across the ψ_branch_ range measured (Figure 3a). Evaluating the occurrence of increases in ψ_branch_ (i.e., positive Δψ_branch_) with regards to key hydraulic thresholds showed that 64.30% of increases occurred between the ψ_branch_ corresponding to branch *P*_12_‐*P*_88_. With regards to the leaf embolism thresholds, 41.90% of the ψ_branch_ increases occurred between leaf *P*_12_–*P*_88_ and were strongly clustered around leaf *P*_50_, as 18.88% occurred within a ± 0.1 MPa interval around leaf *P*_50_ (Figure 3b). Increases in ψ_branch_ were slightly less clustered around branch *P*_50_, as 16.03% of the increases occurred within a ± 0.1 MPa interval around branch *P*_50_ (Figure 3c). Despite the variability in hydraulic thresholds across species, the increases in ψ_branch_ relative to the species‐specific thresholds systematically showed the same patterns described above for each of the five species (Figure S5).

**Figure 3:**
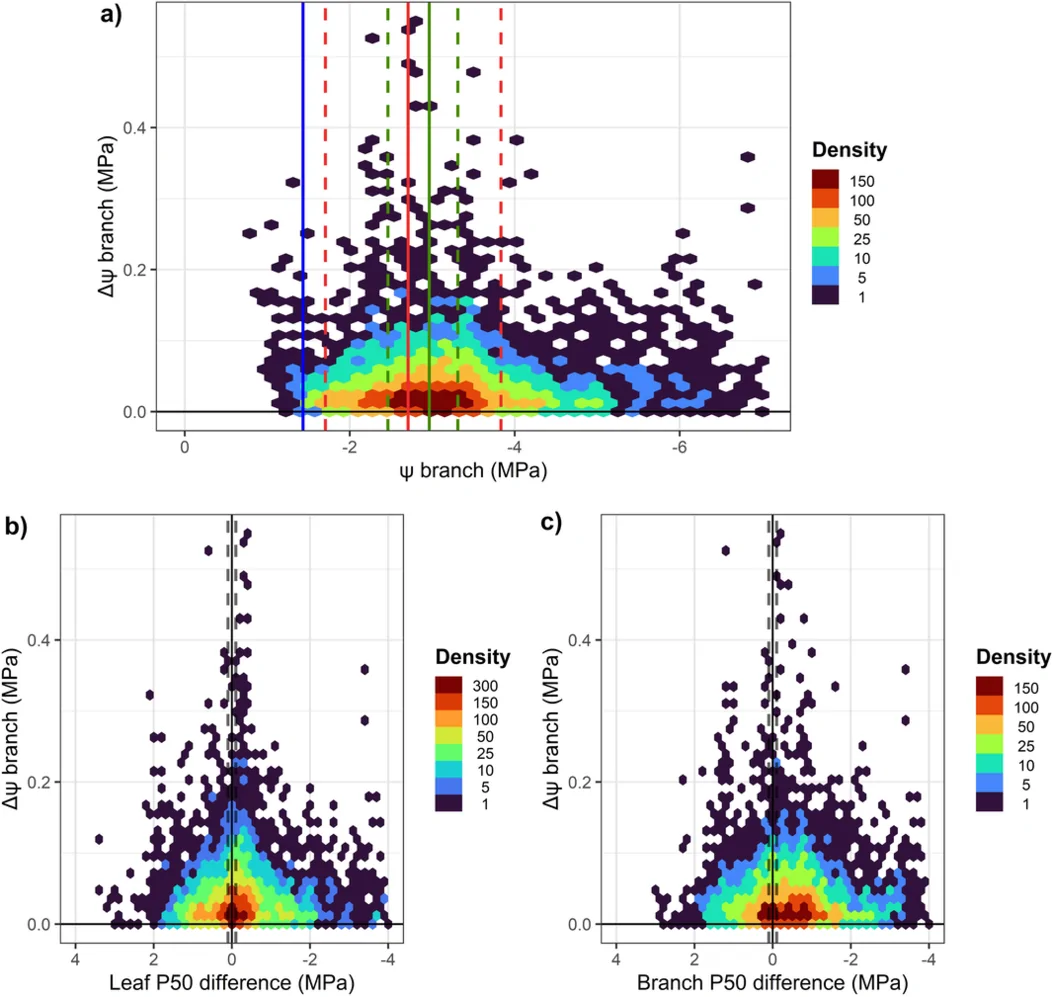
Increases in ψ_branch_ over time (i.e., positive Δψ_branch_), measured every 10 min, plotted against ψ_branch_ for all 75 curves (a). The colour of points represents the density of observations. The vertical lines represent the mean hydraulic thresholds across the five study species (i.e., mean across all 75 branches), where blue = TLP; red solid = branch P_50_; red dashed = branch P_12_ and P_88_; green solid = leaf P_50_; green dashed = leaf P_12_ and P_88_. The results in (a) show that the majority of increases in ψ_branch_ occurred within the branch embolism range (i.e., between branch P_12_–P_88_). Increases in ψ_branch_ are also plotted against the difference between leaf P_50_ and the ψ_branch_ at which the respective increase in ψ_branch_ occurred (b), with positive values along the x‐axis indicating that the increase in ψ_branch_ occurred before leaf P_50_. This difference was evaluated within each individual curve using the curve‐specific leaf P_50_. Dashed lines represent a ± 0.1 MPa interval around leaf P_50_. The results in (b) show that increases in ψ_branch_ are strongly clustered around leaf P_50_, which lies within the branch embolism range, and that the magnitude of increased ψ_branch_ is also greatest in this range. The high density of observations within the narrow interval of ψ_branch_ around leaf P_50_, illustrated by the dark red points, also indicates that ψ_branch_ stayed within the leaf P_50_ range for a very long time. For comparison, increases in ψ_branch_ are also plotted against their difference with branch P_50_ in (c). [Color figure can be viewed at wileyonlinelibrary.com]

### 3.3 Is the Water Released From Vessel Embolism Likely to Be Physiologically Relevant?

The water content inside woody vessels was estimated for 72 out of 75 branches based on anatomical features. Model parameters and equations are shown in Table S2. Vessel diameter at 1 m distance from the tip of branches was 71.91 ± 14.43 µm on average (mean ± SD) and ranged from 40.98 to 106.31 µm (Figure 4a). Vessel lumen fraction averaged 0.16 ± 0.03 (mean ± SD), with a range of 0.07–0.24 (Figure 4b). The estimated mean vessel water content for branch segments of 1 m length was 12.55 ± 2.32 g (mean ± 95% CI) and ranged from 1.41 to 51.25 g. Assuming all of the water released from woody vessel embolism eventually ended up in the leaves, the water released at 50% vessel embolism (Figure 4c) would increase leaf water potential (ψ_leaf_) by 2.05 ± 0.40 MPa on average (mean ± 95% CI; Figure 4d). At 12% and 88% vessel embolism, ψ_leaf_ was predicted to increase by 0.49 ± 0.10 MPa and 3.60 ± 0.71 MPa on average (mean ± 95% CI), respectively.

**Figure 4:**
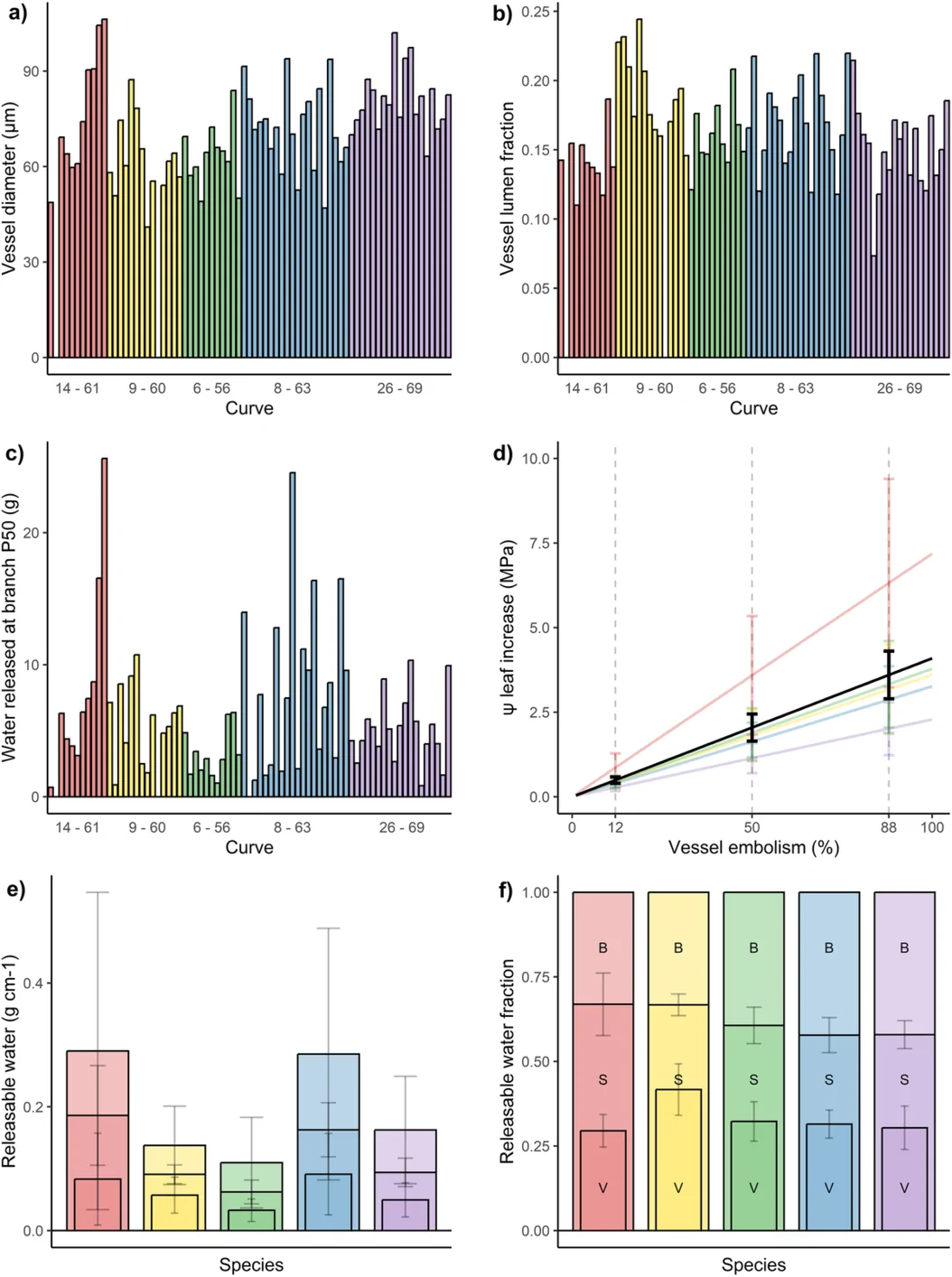
Woody xylem vessel diameter (µm) and lumen fraction (unitless), measured at 1 m distance from the tip of branches (a, b), plotted for each sample (*n* = 73). Red = *Dipterocarpus caudiferus*, yellow = *Dryobalanops lanceolata*, green = *Parashorea malaanonan*, blue = *Parashorea tomentella*, purple = *Rubroshorea johorensis*. Bars within species are in order of increasing branch sampling height. Minimum and maximum height (m) within species are displayed along the *x*‐axis. The estimated amount of water released from woody vessels at 50% embolism is shown for 72 samples (c). Colours and order of bars are as above. The estimated increase in ψ_leaf_ as a result of the water released from woody vessel embolism is plotted against the percentage of vessel embolism for each species (d). Colours are as above; black = mean trendline across species (*n* = 72); error bars represent the 95% confidence intervals. The total water that can be released from the sapwood and bark during desiccation up to P_50_ is shown for each species (mean ± SD) as a stacked bar graph, accounting for the respective tissue volumes per cm of branch length in each of our sampled branches (*n* = 66), measured at 1 m distance from the tip of branches (e). The top and bottom part of each bar represents the contribution of bark (B) and sapwood (S), respectively (mean ± SD). The contribution of vessels (V) specifically (mean ± SD), which are included in the sapwood, is plotted within the sapwood portion for each bar. The relative contribution (mean ± SD) of these tissues to total releasable water, termed ‘releasable water fraction’ (unitless), is shown in (f). Colours of bars are as above. [Color figure can be viewed at wileyonlinelibrary.com]

Lastly, for 66 sampled branches, we derived the ‘standardised water content’ (i.e., the water content of the sapwood and bark combined per cm of branch length; g cm^−1^; Figure S6a) from measurements of saturated water content in the sapwood and bark at 1 m distance from the tip of branches. These values were compared with our estimates of water content in the vessels of the sapwood (Figure S6). The relative contribution of bark, sapwood and vessels specifically to the standardised water content, termed ‘standardised water content fraction’, is shown in Figure S6b. The total water likely to be released from these tissues during desiccation up to *P*_50_ is shown in Figure 4e. This analysis revealed that vessel water content significantly contributes to the total water released from branch tissues during desiccation (Figure 4f). Across all branches, the contribution of vessel water content to the total releasable water up to *P*_50_, termed ‘releasable water fraction’ (unitless), was 0.33 ± 0.07 on average (mean ± SD) and ranged from 0.20 to 0.53 (Figure 4f). In comparison, the contribution of bark and non‐vessel sapwood was 0.39 ± 0.08 and 0.28 ± 0.07 on average (mean ± SD), respectively (Figure 4f).

## 4 Discussion

Leaf vulnerability curves constructed using high temporal resolution measurements reveal that increases in branch water potential (ψ_branch_) during desiccation are a common phenomenon across all five dipterocarp species studied. In this study, we show that increases in ψ_branch_ reached 0.036 MPa on average and temporarily prevented further declines in ψ_branch_. Although the magnitude and variability of these increases varied both across and within species, our results show that increases in ψ_branch_ can be substantial and halt further declines in ψ_branch_ for considerable periods of time. This is, to our knowledge, the first time that the occurrence of increased ψ_branch_ during periods of embolism has been reported for tropical trees. If this mechanism allows these trees to reduce the risk of further embolism, it may allow them to prevent catastrophic embolism during periods of increased water stress, potentially making them more resistant to drought than previously thought (Hölttä et al. 2009; Scholz et al. 2011; Bennett et al. 2015; Liu et al. 2022). This is critical as these species represent some of the largest tropical species in the world, which play a key role in forest ecosystems (Lindenmayer et al. 2012) and are critical for maintaining a global carbon store (Lutz et al. 2012; Qie et al. 2017) and source of biodiversity (Brearley et al. 2016).

### 4.1 Dynamic Changes in Branch Water Potential During Desiccation

Our study demonstrated that every species and branch examined experienced periods of positive change in ψ_branch_ during desiccation. Given that no additional supply of water was available to the plants during desiccation, this water must have come from internal plant sources, most likely from water redistributed as vessels embolised (Tyree and Yang 1990; Tyree and Zimmermann 2002). Thus, while embolism results in a loss of hydraulic conductivity and is not costless (as embolised xylem must be repaired or replaced), our results indicate that it can affect plant water status positively in the short term due to its capacitive effect (Hölttä et al. 2009; Gleason et al. 2014) and may therefore act, within limits, as a water deficit buffering mechanism (Tyree and Yang 1990; Lo Gullo and Salleo 1992). It is also likely that a recently embolised vessel, which has a gas pressure close to zero (i.e., vacuum), will attract gas by diffusion from surrounding tissue (Wang, Liu, et al. 2015; Wang, Pan, et al. 2015). This local extraction is likely to include gas dissolved in xylem sap of vessels surrounding the recently embolised vessel. As such, changes in gas concentration and pressure could provide an additional buffering effect, assuming that the amount of gas relates to embolism spread (Guan et al. 2021; Avila et al. 2022, 2023; Silva et al. 2024).

Our results show that increases in ψ_branch_ mostly occurred within the *P*_12_–*P*_88_ embolism range of branches and were almost completely absent outside of this range. Given the ψ_branch_ range where the increases occurred and the tight correlation between the magnitude and frequency of ψ_branch_ increases with branch embolism thresholds (e.g., *P*_50_, Figure 3), our results indicate that the observed increases in ψ_branch_ are most likely driven by the release of water from embolised vessels. However, we cannot fully exclude that water released from other sources, such as parenchyma cells or the bark, may also be involved, as these can influence branch desiccation rate and therefore the timing and magnitude of the observed increases. Despite considerable differences in hydraulic thresholds among species, the patterns of ψ_branch_ increases were consistent across the five species studied, suggesting that this may be a common phenomenon within the Dipterocarpaceae family.

Interestingly, in all species, the peak magnitude and frequency of increases in ψ_branch_ were strongly centred around leaf *P*_50_ (Figure 3). This suggests that the buffering effect associated with embolism formation would be greatest when vessels in leaf and branch xylem embolise simultaneously at maximum rates (i.e., during the steepest part of the vulnerability curve, around *P*_50_). The clustering of increases in ψ_branch_ around leaf *P*_50_ (Figure 3) also suggests that, alongside branch capacitance, leaf capacitance could play an important role in avoiding critical thresholds for hydraulic failure. These results support modelling simulations that have demonstrated that feedback from embolising xylem vessels can theoretically hold xylem water potential around the *P*_50_ mark for considerable periods of time (Hölttä et al. 2009). Similarly, Sevanto et al. (2014) reported that ψ_branch_ remained near homeostasis in the −2 to −3 MPa range, most likely due to water release from embolising needle xylem. Although leaves and branches in our study exhibited similar embolism resistance (Figure 3a), significant differences between leaf and branch *P*_50_ were observed for some, but not all species. Additionally, branches reached *P*_50_ 0.26 MPa before leaves on average. Although these results cannot definitively rule out hydraulic segmentation between leaves and branches, they provide little support for the hypothesis that leaves in the species studied are being sacrificed (i.e., by embolising first) to protect the costly woody xylem (Levionnois et al. 2020; Rimer et al. 2026). Clearly, further investigation into how water release from embolism relates to hydraulic segmentation and, more broadly, plant drought‐resistance strategies remains an important avenue for future research.

Our results indicate that the timing and frequency of increases in ψ_branch_ may provide an important buffer to reduce the risk of further embolism, at least temporarily (Hölttä et al. 2009; Scholz et al. 2011), before more lethal thresholds of embolism are reached, like *P*_88_ (Urli et al. 2013). Furthermore, it has recently been suggested that the spread of embolism through the remaining xylem is faster once a certain embolism threshold has been passed (i.e., when a substantial portion of gas has entered the xylem tissue; Avilla et al. 2022). This, together with the observed scaling between increased ψ_branch_ and ongoing embolism, would suggest that the buffering effect becomes increasingly important as embolism progresses, potentially providing an additional layer of protection against reaching critical embolism thresholds and a rapid escalation of hydraulic failure.

Several studies have successfully used psychrometers to construct leaf vulnerability curves (Brodribb, Bienaimé, et al. 2016; Skelton et al. 2017; Lucani 2019; Creek et al. 2020; Powers et al. 2020; Yao et al. 2021; Ziegler et al. 2023). Yet, studies on tropical trees remain rare (Powers et al. 2020; Ziegler et al. 2023) and the occurrence of increased ψ_branch_ during periods of embolism has, to our knowledge, never been reported. In temperate systems, however, there are some examples of increased water potentials. Lo Gullo and Salleo (1992) reported that water from embolised vessels rehydrated leaves of dehydrated *Populus deltoides* twigs. Additionally, Beckett (1997) detected increases in ψ_leaf_ in some species, while using psychrometers to construct pressure–volume curves of lichens, bryophytes, ferns and angiosperms, although these increases occurred at much higher water potentials (meaning less negative) and were attributed to the presence of negative turgor. It remains unclear why increases in ψ_branch_ were observed in all five of our study species, because similar observations have not been reported in other studies (Lamarque et al. 2018; Avilla et al. 2022). It may be that the increases are particularly large in our species, making them easier to separate from potential errors that can make it hard to detect increased ψ_branch_, or it may be that the phenomena we observed are restricted to this tropical tree family.

Artefactual detection of increased ψ_branch_ may potentially occur from the complexity of using psychrometers, particularly at high temporal resolution (Vergeynst et al. 2015). Changes in microclimate leading to temperature gradients in the psychrometer during the experiment or electronic noise may cause increases in the ψ_branch_ reported by psychrometers (Comstock 2000). However, the duration and patterns of occurrence of the increases in ψ_branch_ across all branches make this unlikely to be the cause of our results. First, the increases in ψ_branch_ were not significantly correlated with changes in temperature or relative humidity in the lab, and so were unrelated to changes in microclimate (Figures S2 and S3). Second, if electronic noise alone was the source of our results, the increases in ψ_branch_ would be stochastic and not clustered around particular parts of the curve. Furthermore, they would not occur consecutively over time, as observed here. Also, for electronic noise to be the cause, the variability of increased ψ_branch_ across curves would be low and the mean increase across curves should be more constant than we observed (Figures 1 and 2).

Consequently, we do not suspect that our results are artefacts, but believe they are related to phenomena affecting the water status within the plant. We note that while additional verification of the psychrometer water potential measurements throughout the experiments with a pressure chamber would have been ideal, this was not logistically possible at the time of our study. However, even with perfect accuracy, the magnitude of measured water potential increases will still be influenced by the sample desiccation rate, which our study could not fully standardise due to anatomical and structural variation across branch samples. Because of this, our study does not intend to provide perfect quantitative estimates of the increases, but rather to document and highlight the frequent occurrence of these unexpected rises in water potential. While we acknowledge the limitations regarding measurement accuracy, inherently tied to psychrometer use, we believe the strength of our study lies in drawing attention to the phenomenon of increasing water potential during desiccation, which could be more common in these tropical species than previously assumed. Our goal is therefore not to argue that embolism‐driven water potential increases are universal, but to highlight that they may occur under certain conditions and within at least some species, and thus merit further investigation. We also stress that the primary focus still remains the underlying mechanism, namely the ability of embolism‐associated water release to temporarily buffer changes, and thus prevent further declines, in plant water potential under field conditions, rather than the magnitude of the increases per se.

### 4.2 The Capacitive Effect of Embolism as a Crucial Component of Whole‐Tree Water Status

How much water is released to the plant during embolism and the fate of this water are ultimately very challenging to observe directly. Here, we theoretically modelled, using xylem anatomical data collected on each of our branches, the water that could potentially be released from vessels during branch embolism (Figure 4c). Furthermore, our results reveal that woody vessel water content contributes significantly to the total releasable water from branch tissues during desiccation up to *P*_50_, accounting for 33.0 ± 7.2% on average (mean ± SD) and up to 52.8% (Figure 4f). This highlights the potentially significant role of water released from vessel embolism for plant water status. However, it is important to note that these estimates are for cut branches. In reality, trees also have access to soil water reserves and their total xylem volume is much larger than that of our 1‐m‐long theoretical branches. If the entire tree is taken into account, the total amount of water released and its buffering effect on tree water status may be much larger. Additionally, our estimates also do not take into account the water that may be released from other tissues, such as tracheids, parenchyma, fibres or the bark, which can constitute large tissue fractions in tropical trees (Ziemińska et al. 2015; Morris et al. 2016; Lourenço et al. 2022;) and have been shown to contribute substantially to water storage capacity and capacitance (Holbrook 1995; Borchert and Pockman 2005; Pfautsch et al. 2015; Jupa et al. 2016; Knipfer et al. 2019). Assuming that 20% of the bark and non‐vessel sapwood water content can be released up to *P*_50_, the relative contribution of the bark and non‐vessel sapwood to the total releasable water was 38.6 ± 7.6% and 28.4 ± 6.7% on average (mean ± SD), respectively (Figure 4f). These findings indicate a large capacity for water storage in these tissues and suggest that their role in whole‐tree capacitance should not be overlooked.

Previous studies have shown that bark tissues release and replenish water on a diurnal basis (De Schepper et al. 2012) and that ray parenchyma plays a crucial role in the exchange of water between bark and xylem tissues (Pfautsch et al. 2015). Water release from the bark likely occurs at less negative water potentials than those causing water depletion of the xylem matrix (i.e., non‐vessel sapwood) and vessel embolism, and may therefore be particularly useful to reduce the risk of hydraulic failure during early drought (Pfautsch et al. 2015). Additionally, in a study on intact saplings of American chestnut, Knipfer et al. (2019) found that most of the water stored in the xylem matrix surrounding the vessels was released after vessel embolism began, suggesting that this release likely plays a role in regulating tree water status during drought, but is not primarily aimed at preventing vessel embolism. More research is needed here to determine whether the findings by Knipfer et al. (2019) also hold for mature trees growing in tropical environments. Tropical hardwoods, which often contain greater volumes of parenchyma tissue relative to temperate species (Ziemińska et al. 2015; Morris et al. 2016), may have unique adaptations that influence how water is stored and released. The prevalence of paratracheal parenchyma in tropical species, including in all of the species studied, and more broadly, within the Dipterocarpaceae family (Ashton 2003), could suggest different mechanisms of water release under drought stress compared to temperate species. If and to what extent these additional sources of water release may have contributed to the observed increases in ψ_branch_ is not possible to tell with certainty from our experiments, and their role in capacitance and tree water status should be explored in more detail.

Depending on the amount of water that is released from embolised vessels and where it is redistributed to, it could significantly impact the water balance of trees (Sperry et al. 2008; Scholz et al. 2011). This may allow trees to reduce the overall damage to the hydraulic system experienced during periods of increased water stress, as less xylem would need to be repaired or replaced if temporary increases in water potential can slow the rate of further embolism. Although it remains challenging to predict the amount and consequences of the water released in non‐excised branches under natural conditions—and uncertainty remains about how much of this water reaches the leaves—our results obtained from high temporal resolution leaf vulnerability curves shed light onto a novel mechanism by which increased ψ_branch_ may temporarily mitigate further embolism and preserve hydraulic function in leaves (Figures 1 and S4). Extending this approach to woody xylem vulnerability curves with comparable temporal resolution in future studies will be crucial for assessing the resilience of woody tissues and the overall integrity of the plant hydraulic system during drought. Fully understanding the impact of water released from vessel embolism on subsequent embolism events likely requires simultaneous high temporal resolution measurements of both water potential and embolism formation in both leaves and branches. This is likely the only way to determine whether transient increases in water potential are directly linked to embolism dynamics within and between organs. Moreover, further research is needed to more precisely quantify the redistribution of water from branches to leaves. Such studies will be critical for determining both the amount and rate at which water released from vessel embolism becomes available to leaves. Furthermore, determining how water redistribution between plant organs relates to hydraulic segmentation between leaves and branches (Rimer et al. 2026), while also considering additional mechanisms that shape plant water status during desiccation, such as residual conductance from leaf and bark (Burlett et al. 2025), is also an important area for future studies.

Finally, as tree water storage capacity and the capacitive effect of embolism are expected to increase with tree size (Phillips et al. 2003; Hölttä et al. 2009; Scholz et al. 2011), this mechanism could be particularly important for alleviating drought in these tall dipterocarps, and might contribute towards the extraordinary growth and maximum tree sizes in the Dipterocarpaceae family. However, the relationship between increased ψ_branch_ and height was significant for only one species, perhaps suggesting that this mechanism is not related only to tree height, potentially due to exceptionally large capacitance or water storage capacity within the tree crown (Williams et al. 2017). Our study shows that significantly more research in this area may help us understand how these forests manage to survive periods of drought and maintain such colossal sizes.

## 5 Conclusion

Our findings reveal that isovolumetric water potential increases, which refers to increasing plant water potential without an increase in water volume, can occur during the desiccation of dipterocarp trees. These increases do not occur randomly, but rather when plants are in the water potential range where embolism occurs, and are sufficiently large to be physiologically relevant for these trees to mitigate periods of increased water stress. Our process modelling parametrised from anatomical measurements indicates that the release of water stored in the xylem significantly contributes to the isovolumetric water potential increases observed from branch samples sampled from the field, corroborating our hypothesis that the phenomenon we observed using psychrometers is most likely driven by water released from the xylem during embolism.

Traditionally, drought vulnerability has been evaluated by looking solely at *P*_50_, focusing on a tree's ability to avoid severe levels of embolism. However, our study suggests that water in the xylem may be more dynamic than previously assumed, and that mechanisms delaying or mitigating high levels of embolism might have an important role in plant drought resistance strategies. This capacity may be linked to the ability of some species in the Dipterocarpaceae family to grow so large, or to survive periods of severe drought. However, the extent to which this buffering capacity is common amongst other tropical or temperate species remains to be explored in greater detail. Future assessments of vulnerability to drought should therefore not only consider the avoidance of severe embolism, but also examine how and when trees can sustain hydraulic function under such conditions. This broader approach will provide a more comprehensive understanding of tree water dynamics and their strategies for coping with drought.

## Conflicts of Interest

The authors declare no conflicts of interest.

## Supporting information

Supporting File 1

Supporting File 2

## Data Availability

The data supporting the findings of this study are available within the paper and supplementary files.

## References

[pce70647-bib-0001] Anderegg, W. R. L. , A. Flint , C. Huang , et al. 2015. “Tree Mortality Predicted From Drought‐Induced Vascular Damage.” Nature Geoscience 8, no. 5: 367–371.

[pce70647-bib-0002] Ashton, P. S. 2003. “Dipterocarpaceae.” In Flowering Plants· Dicotyledons: Malvales, Capparales and Non‐betalain Caryophyllales, edited by K. Kubitzki and C. Bayer , 182–197. Springer Berlin Heidelberg.

[pce70647-bib-0003] Avilla, R. T. , X. Guan , C. N. Kane , et al. 2022. “Xylem Embolism Spread Is Largely Prevented by Interconduit Pit Membranes Until the Majority of Conduits Are Gas‐Filled.” Plant, Cell & Environment 45, no. 4: 1204–1215. 10.1111/pce.14253 34984700

[pce70647-bib-0004] Avilla, R. T. , C. N. Kane , T. A. Batz , et al. 2023. “The Relative Area of Vessels in Xylem Correlates With Stem Embolism Resistance Within and Between Genera.” Tree Physiology 43, no. 1: 75–87. 10.1093/treephys/tpac110 36070431

[pce70647-bib-0005] Banin, L. , T. R. Feldpausch , O. L. Phillips , et al. 2012. “What Controls Tropical Forest Architecture? Testing Environmental, Structural and Floristic Drivers.” Global Ecology and Biogeography 21, no. 12: 1179–1190.

[pce70647-bib-0006] Banin, L. , S. L. Lewis , G. Lopez‐Gonzalez , et al. 2014. “Tropical Forest Wood Production: A Cross‐Continental Comparison.” Journal of Ecology 102, no. 4: 1025–1037.

[pce70647-bib-0007] Beckett, R. 1997. “Pressure–Volume Analysis of a Range of Poikilohydric Plants Implies the Existence of Negative Turgor in Vegetative Cells.” Annals of Botany 79, no. 2: 145–152.

[pce70647-bib-0008] Bennett, A. C. , N. G. McDowell , C. D. Allen , and K. J. Anderson‐Teixeira . 2015. “Larger Trees Suffer Most During Drought in Forests Worldwide.” Nature Plants 1, no. 10: 15139. 10.1038/nplants.2015.139 27251391

[pce70647-bib-0009] Bittencourt, P. R. L. , D. C. Bartholomew , L. F. Banin , et al. 2022. “Divergence of Hydraulic Traits Among Tropical Forest Trees Across Topographic and Vertical Environment Gradients in Borneo.” New Phytologist 235, no. 6: 2183–2198. 10.1111/nph.18280 PMC9545514 35633119

[pce70647-bib-0010] Bittencourt, P. , A. Scheire , P. Jotan , et al. 2026. Height Does not Impair the Hydraulic System of the Tallest Tropical Dipterocarp Trees. *Science*. 10.1126/science.aea3911. 42391372

[pce70647-bib-0011] Blackman, C. J. , T. J. Brodribb , and G. J. Jordan . 2009. “Leaf Hydraulics and Drought Stress: Response, Recovery and Survivorship in Four Woody Temperate Plant Species.” Plant, Cell & Environment 32, no. 11: 1584–1595. 10.1111/j.1365-3040.2009.02023.x 19627564

[pce70647-bib-0012] Blackman, C. J. , T. J. Brodribb , and G. J. Jordan . 2010. “Leaf Hydraulic Vulnerability Is Related to Conduit Dimensions and Drought Resistance Across a Diverse Range of Woody Angiosperms.” New Phytologist 188, no. 4: 1113–1123. 10.1111/j.1469-8137.2010.03439.x 20738785

[pce70647-bib-0013] Blackman, C. J. , S. M. Gleason , Y. Chang , A. M. Cook , C. Laws , and M. Westoby . 2014. “Leaf Hydraulic Vulnerability to Drought Is Linked to Site Water Availability Across a Broad Range of Species and Climates.” Annals of Botany 114, no. 3: 435–440. 10.1093/aob/mcu131 PMC4204664 25006181

[pce70647-bib-0014] Borchert, R. , and W. T. Pockman . 2005. “Water Storage Capacitance and Xylem Tension in Isolated Branches of Temperate and Tropical Trees.” Tree Physiology 25, no. 4: 457–466. 10.1093/treephys/25.4.457 15687094

[pce70647-bib-0015] Brearley, F. Q. , L. F. Banin , and P. Saner . 2016. “The Ecology of the Asian Dipterocarps.” Plant Ecology & Diversity 9, no. 5–6: 429–436.

[pce70647-bib-0016] Brodribb, T. J. , D. Bienaimé , and P. Marmottant . 2016. “Revealing Catastrophic Failure of Leaf Networks under Stress.” Proceedings of the National Academy of Sciences 113, no. 17: 4865–4869. 10.1073/pnas.1522569113 PMC4855591 27071104

[pce70647-bib-0017] Brodribb, T. J. , D. J. M. S. Bowman , S. Nichols , S. Delzon , and R. Burlett . 2010. “Xylem Function and Growth Rate Interact to Determine Recovery Rates After Exposure to Extreme Water Deficit.” New Phytologist 188, no. 2: 533–542. 10.1111/j.1469-8137.2010.03393.x 20673281

[pce70647-bib-0018] Brodribb, T. J. , M. Carriqui , S. Delzon , and C. Lucani . 2017. “Optical Measurement of Stem Xylem Vulnerability.” Plant Physiology 174, no. 4: 2054–2061. 10.1104/pp.17.00552 PMC5543975 28684434

[pce70647-bib-0019] Brodribb, T. J. , and H. Cochard . 2009. “Hydraulic Failure Defines the Recovery and Point of Death in Water‐Stressed Conifers.” Plant Physiology 149, no. 1: 575–584. 10.1104/pp.108.129783 PMC2613726 19011001

[pce70647-bib-0020] Brodribb, T. J. , and N. M. Holbrook . 2003. “Stomatal Closure During Leaf Dehydration, Correlation With Other Leaf Physiological Traits.” Plant Physiology 132, no. 4: 2166–2173. 10.1104/pp.103.023879 PMC181300 12913171

[pce70647-bib-0021] Brodribb, T. J. , and N. M. Holbrook . 2006. “Declining Hydraulic Efficiency as Transpiring Leaves Desiccate: Two Types of Response.” Plant, Cell & Environment 29, no. 12: 2205–2215. 10.1111/j.1365-3040.2006.01594.x 17081253

[pce70647-bib-0022] Brodribb, T. J. , R. P. Skelton , S. A. McAdam , D. Bienaimé , C. J. Lucani , and P. Marmottant . 2016. “Visual Quantification of Embolism Reveals Leaf Vulnerability to Hydraulic Failure.” New Phytologist 209, no. 4: 1403–1409. 10.1111/nph.13846 26742653

[pce70647-bib-0023] Burlett, R. , S. Trueba , X. P. Bouteiller , et al. 2025. “Minimum Leaf Conductance During Drought: Unravelling Its Variability and Impact on Plant Survival.” New Phytologist 246, no. 3: 1001–1014. 10.1111/nph.70052 PMC11982793 40059431

[pce70647-bib-0024] Choat, B. , T. J. Brodribb , C. R. Brodersen , R. A. Duursma , R. López , and B. E. Medlyn . 2018. “Triggers of Tree Mortality Under Drought.” Nature 558, no. 7711: 531–539. 10.1038/s41586-018-0240-x 29950621

[pce70647-bib-0025] Choat, B. , S. Jansen , T. J. Brodribb , et al. 2012. “Global Convergence in the Vulnerability of Forests to Drought.” Nature 491, no. 7426: 752–755. 10.1038/nature11688 23172141

[pce70647-bib-0026] Cochard, H. , E. Badel , S. Herbette , S. Delzon , B. Choat , and S. Jansen . 2013. “Methods for Measuring Plant Vulnerability to Cavitation: A Critical Review.” Journal of Experimental Botany 64, no. 15: 4779–4791. 10.1093/jxb/ert193 23888067

[pce70647-bib-0027] Comstock, J. P. 2000. “Correction of Thermocouple Psychrometer Readings for the Interaction of Temperature and Actual Water Potential.” Crop Science 40, no. 3: 709–712.

[pce70647-bib-0028] Creek, D. , L. J. Lamarque , J. M. Torres‐Ruiz , et al. 2020. “Xylem Embolism in Leaves Does Not Occur With Open Stomata: Evidence From Direct Observations Using the Optical Visualization Technique.” Journal of Experimental Botany 71, no. 3: 1151–1159. 10.1093/jxb/erz474 31641746

[pce70647-bib-0029] Dixon, M. , and A. Downey . 2017. PSY1 Stem Psychrometer Manual. ICT International.

[pce70647-bib-0030] Duursma, R. , and B. Choat . 2017. “Fitplc: An R Package to Fit Hydraulic Vulnerability Curves.” Journal of Plant Hydraulics 4: e002.

[pce70647-bib-0031] Duursma, R. A. , C. J. Blackman , R. Lopéz , N. K. Martin‐StPaul , H. Cochard , and B. E. Medlyn . 2019. “On the Minimum Leaf Conductance: Its Role in Models of Plant Water Use, and Ecological and Environmental Controls.” New Phytologist 221, no. 2: 693–705. 10.1111/nph.15395 30144393

[pce70647-bib-0032] Estoque, R. C. , M. Ooba , V. Avitabile , et al. 2019. “The Future of Southeast Asia's Forests.” Nature Communications 10, no. 1: 1829. 10.1038/s41467-019-09646-4 PMC6478739 31015425

[pce70647-bib-0033] Fox, J. E. D. 1972. The Natural Vegetation of the Sabah and Natural Regeneration of the Dipterocarp Forests. Bangor University.

[pce70647-bib-0034] Gatti, L. V. , L. S. Basso , J. B. Miller , et al. 2021. “Amazonia as a Carbon Source Linked to Deforestation and Climate Change.” Nature 595, no. 7867: 388–393. 10.1038/s41586-021-03629-6 34262208

[pce70647-bib-0035] Gleason, S. M. , C. J. Blackman , A. M. Cook , C. A. Laws , and M. Westoby . 2014. “Whole‐Plant Capacitance, Embolism Resistance and Slow Transpiration Rates All Contribute to Longer Desiccation Times in Woody Angiosperms From Arid and Wet Habitats.” Tree Physiology 34, no. 3: 275–284. 10.1093/treephys/tpu001 24550089

[pce70647-bib-0036] Guan, X. , L. Pereira , S. McAdam , K. F. Cao , and S. Jansen . 2021. “No Gas Source, No Problem: Proximity to Pre‐Existing Embolism and Segmentation Affect Embolism Spreading in Angiosperm Xylem by Gas Diffusion.” Plant, Cell & Environment 44, no. 5: 1329–1345. 10.1111/pce.14016 33529382

[pce70647-bib-0037] Guan, X. , J. Werner , K. F. Cao , et al. 2022. “Stem and Leaf Xylem of Angiosperm Trees Experiences Minimal Embolism in Temperate Forests During Two Consecutive Summers With Moderate Drought.” Plant Biology 24, no. 7: 1208–1223. 10.1111/plb.13384 34990084

[pce70647-bib-0038] Holbrook, N. M. 1995. “Stem Water Storage.” In Plant Stems, edited by B. L. Gartner , 151–174. Academic Press.

[pce70647-bib-0039] Hölttä, T. , H. Cochard , E. Nikinmaa , and M. Mencuccini . 2009. “Capacitive Effect of Cavitation in Xylem Conduits: Results From a Dynamic Model.” Plant, Cell & Environment 32, no. 1: 10–21. 10.1111/j.1365-3040.2008.01894.x 19076529

[pce70647-bib-0040] Hubau, W. , S. L. Lewis , O. L. Phillips , et al. 2020. “Asynchronous Carbon Sink Saturation in African and Amazonian Tropical Forests.” Nature 579, no. 7797: 80–87. 10.1038/s41586-020-2035-0 PMC7617213 32132693

[pce70647-bib-0041] ICT International . 2014. PSY1 Stem Psychrometer: Quick Start Guide. ICT International.

[pce70647-bib-0042] IPCC . 2022. Climate Change 2022: Impacts, Adaptation, and Vulnerability. Contribution of Working Group II to the Sixth Assessment Report of the Intergovernmental Panel on Climate Change, Cambridge University Press. 10.1017/9781009325844.

[pce70647-bib-0043] Johnson, D. M. , K. A. McCulloh , D. R. Woodruff , and F. C. Meinzer . 2012. “Hydraulic Safety Margins and Embolism Reversal in Stems and Leaves: Why Are Conifers and Angiosperms so Different?” Plant Science 195: 48–53. 10.1016/j.plantsci.2012.06.010 22920998

[pce70647-bib-0045] Jucker, T. , B. Bongalov , D. F. R. P. Burslem , et al. 2018. “Topography Shapes the Structure, Composition and Function of Tropical Forest Landscapes.” Ecology Letters 21, no. 7: 989–1000. 10.1111/ele.12964 PMC6849614 29659115

[pce70647-bib-0046] Jupa, R. , L. Plavcová , V. Gloser , and S. Jansen . 2016. “Linking Xylem Water Storage With Anatomical Parameters in Five Temperate Tree Species.” Tree Physiology 36, no. 6: 756–769. 10.1093/treephys/tpw020 27083523

[pce70647-bib-0047] Knipfer, T. , C. Reyes , J. M. Earles , et al. 2019. “Spatiotemporal Coupling of Vessel Cavitation and Discharge of Stored Xylem Water in a Tree Sapling.” Plant Physiology 179, no. 4: 1658–1668. 10.1104/pp.18.01303 PMC6446773 30718351

[pce70647-bib-0048] Lamarque, L. J. , D. Corso , J. M. Torres‐Ruiz , et al. 2018. “An Inconvenient Truth About Xylem Resistance to Embolism in the Model Species for Refilling *Laurus nobilis* L.” Annals of Forest Science 75, no. 3: 88.

[pce70647-bib-0049] Levionnois, S. , C. Ziegler , S. Jansen , et al. 2020. “Vulnerability and Hydraulic Segmentations at the Stem–Leaf Transition: Coordination Across Neotropical Trees.” New Phytologist 228, no. 2: 512–524. 10.1111/nph.16723 32496575

[pce70647-bib-0050] Lindenmayer, D. B. , W. F. Laurance , and J. F. Franklin . 2012. “Global Decline in Large Old Trees.” Science 338, no. 6112: 1305–1306. 10.1126/science.1231070 23224548

[pce70647-bib-0051] Liu, L. , X. Chen , P. Ciais , et al. 2022. “Tropical Tall Forests Are More Sensitive and Vulnerable to Drought Than Short Forests.” Global Change Biology 28, no. 4: 1583–1595. 10.1111/gcb.16017 34854168

[pce70647-bib-0052] Lo Gullo, M. A. , and S. Salleo . 1992. “Water Storage in the Wood and Xylem Cavitation in 1‐Year‐Old Twigs of Populus Deltoides Bartr.” Plant, Cell & Environment 15, no. 4: 431–438.

[pce70647-bib-0053] Lourenço, Jr., J. , B. J. Enquist , G. von Arx , et al. 2022. “Hydraulic Tradeoffs Underlie Local Variation in Tropical Forest Functional Diversity and Sensitivity to Drought.” New Phytologist 234, no. 1: 50–63. 10.1111/nph.17944 34981534

[pce70647-bib-0054] Lucani, C. 2019. “New and Improved Techniques for Assessing Drought Tolerance in Plants.” Doctoral diss., University of Tasmania.

[pce70647-bib-0055] Lutz, J. A. , A. J. Larson , M. E. Swanson , and J. A. Freund . 2012. “Ecological Importance of Large‐Diameter Trees in a Temperate Mixed‐Conifer Forest.” PLoS One 7, no. 5: e36131. 10.1371/journal.pone.0036131 PMC3342248 22567132

[pce70647-bib-0056] McDowell, N. , C. D. Allen , K. Anderson‐Teixeira , et al. 2018. “Drivers and Mechanisms of Tree Mortality in Moist Tropical Forests.” New Phytologist 219, no. 3: 851–869. 10.1111/nph.15027 29451313

[pce70647-bib-0057] McDowell, N. , W. T. Pockman , C. D. Allen , et al. 2008. “Mechanisms of Plant Survival and Mortality During Drought: Why Do Some Plants Survive While Others Succumb to Drought?” New Phytologist 178, no. 4: 719–739. 10.1111/j.1469-8137.2008.02436.x 18422905

[pce70647-bib-0058] Meinzer, F. C. , S. A. James , and G. Goldstein . 2004. “Dynamics of Transpiration, Sap Flow and Use of Stored Water in Tropical Forest Canopy Trees.” Tree Physiology 24, no. 8: 901–909. 10.1093/treephys/24.8.901 15172840

[pce70647-bib-0059] Meinzer, F. C. , D. M. Johnson , B. Lachenbruch , K. A. McCulloh , and D. R. Woodruff . 2009. “Xylem Hydraulic Safety Margins in Woody Plants: Coordination of Stomatal Control of Xylem Tension With Hydraulic Capacitance.” Functional Ecology 23, no. 5: 922–930.

[pce70647-bib-0060] Meinzer, F. C. , D. R. Woodruff , J. C. Domec , et al. 2008. “Coordination of Leaf and Stem Water Transport Properties in Tropical Forest Trees.” Oecologia 156: 31–41. 10.1007/s00442-008-0974-5 18253753

[pce70647-bib-0061] Morris, H. , L. Plavcová , P. Cvecko , et al. 2016. “A Global Analysis of Parenchyma Tissue Fractions in Secondary Xylem of Seed Plants.” New Phytologist 209, no. 4: 1553–1565. 10.1111/nph.13737 PMC5063116 26551018

[pce70647-bib-0062] Nilus, R. 2004. Effect of Edaphic Variation on Forest Structure, Dynamics, Diversity and Regeneration in a Lowland Tropical Rain Forest in Borneo. University of Aberdeen Department of Plant and Soil Science.

[pce70647-bib-0063] Nilus, R. , C. R. Maycock , N. Majalap‐Lee , and D. F. R. P. Burslem . 2011. “Nutrient Limitation of Tree Seedling Growth in Three Soil Types Found in Sabah.” Journal of Tropical Forest Science 23, no. 2: 133–142.

[pce70647-bib-0064] Ogle, K. , J. J. Barber , C. Willson , and B. Thompson . 2009. “Hierarchical Statistical Modeling of Xylem Vulnerability to Cavitation.” New Phytologist 182, no. 2: 541–554. 10.1111/j.1469-8137.2008.02760.x 19210723

[pce70647-bib-0065] Oliveira, R. S. , F. R. C. Costa , E. van Baalen , et al. 2019. “Embolism Resistance Drives the Distribution of Amazonian Rainforest Tree Species Along Hydro‐Topographic Gradients.” New Phytologist 221, no. 3: 1457–1465. 10.1111/nph.15463 30295938

[pce70647-bib-0066] Pammenter, N. W. , and C. Van der Willigen . 1998. “A Mathematical and Statistical Analysis of the Curves Illustrating Vulnerability of Xylem to Cavitation.” Tree Physiology 18, no. 8–9: 589–593. 10.1093/treephys/18.8-9.589 12651346

[pce70647-bib-0067] Pereira, L. , P. Bittencourt , V. S. Pacheco , et al. 2020. “The Pneumatron: An Automated Pneumatic Apparatus for Estimating Xylem Vulnerability to Embolism at High Temporal Resolution.” Plant, Cell & Environment 43, no. 1: 131–142. 10.1111/pce.13647 31461536

[pce70647-bib-0068] Pfautsch, S. , T. Hölttä , and M. Mencuccini . 2015. “Hydraulic Functioning of Tree Stems—Fusing Ray Anatomy, Radial Transfer and Capacitance.” Tree Physiology 35, no. 7: 706–722. 10.1093/treephys/tpv058 26163488

[pce70647-bib-0069] Phillips, N. G. , M. G. Ryan , B. J. Bond , N. G. McDowell , T. M. Hinckley , and J. Cermak . 2003. “Reliance on Stored Water Increases With Tree Size in Three Species in the Pacific Northwest.” Tree Physiology 23, no. 4: 237–245. 10.1093/treephys/23.4.237 12566259

[pce70647-bib-0070] Powers, J. S. , G. Vargas G., T. J. Brodribb , et al. 2020. “A Catastrophic Tropical Drought Kills Hydraulically Vulnerable Tree Species.” Global Change Biology 26, no. 5: 3122–3133. 10.1111/gcb.15037 32053250

[pce70647-bib-0071] Qie, L. , S. L. Lewis , M. J. P. Sullivan , et al. 2017. “Long‐Term Carbon Sink in Borneo's Forests Halted by Drought and Vulnerable to Edge Effects.” Nature Communications 8, no. 1: 1966. 10.1038/s41467-017-01997-0 PMC5736600 29259276

[pce70647-bib-0072] R Core Team . 2020. *R: A Language and Environment for Statistical Computing*. R Foundation for Statistical Computing.

[pce70647-bib-0073] Rimer, I. M. , A. Manandhar , and S. A. McAdam . 2026. “Pre‐Existing Embolism Causes Reverse Vulnerability Segmentation in Populus.” Journal of Experimental Botany 77, no. 6: 1865–1873. 10.1093/jxb/eraf523 41312634

[pce70647-bib-0074] Romell, E. 2011. “Aspects of Regeneration in Tropical Secondary Forests of Northeast Borneo Affected by Selective Logging, El Niño‐Induced Drought and Fire” Swedish University of Agricultural Sciences. https://research.slu.se/en/publications/aspects-of-regeneration-in-tropical-secondary-forests-of-northeas/.

[pce70647-bib-0075] Rowland, L. , A. C. L. da Costa , D. R. Galbraith , et al. 2015. “Death From Drought in Tropical Forests Is Triggered by Hydraulics Not Carbon Starvation.” Nature 528, no. 7580: 119–122. 10.1038/nature15539 26595275

[pce70647-bib-0076] Sack, L. , and N. M. Holbrook . 2006. “Leaf Hydraulics.” Annual Review of Plant Biology 57: 361–381. 10.1146/annurev.arplant.56.032604.144141 16669766

[pce70647-bib-0077] Salomón, R. L. , J. M. Limousin , J. M. Ourcival , J. Rodríguez‐Calcerrada , and K. Steppe . 2017. “Stem Hydraulic Capacitance Decreases With Drought Stress: Implications for Modelling Tree Hydraulics in the Mediterranean Oak Quercus Ilex.” Plant, Cell & Environment 40, no. 8: 1379–1391. 10.1111/pce.12928 28152583

[pce70647-bib-0078] Santiago, L. S. , M. E. De Guzman , C. Baraloto , et al. 2018. “Coordination and Trade‐Offs Among Hydraulic Safety, Efficiency and Drought Avoidance Traits in Amazonian Rainforest Canopy Tree Species.” New Phytologist 218, no. 3: 1015–1024. 10.1111/nph.15058 29457226

[pce70647-bib-0079] Scheire, A. 2025. “Scaling Tropical Giants: How Tree Height Affects the Functioning of Some of the World's Tallest Tropical Rain Forest Species,” PhD thesis, University of Exeter.

[pce70647-bib-0080] De Schepper, V. , D. Van Dusschoten , P. Copini , S. Jahnke , and K. Steppe . 2012. “MRI Links Stem Water Content to Stem Diameter Variations in Transpiring Trees.” Journal of Experimental Botany 63, no. 7: 2645–2653. 10.1093/jxb/err445 22268159

[pce70647-bib-0081] Schneider, C. A. , W. S. Rasband , and K. W. Eliceiri . 2012. “NIH Image to Imagej: 25 Years of Image Analysis.” Nature Methods 9, no. 7: 671–675. 10.1038/nmeth.2089 PMC5554542 22930834

[pce70647-bib-0082] Scholz, F. G. , N. G. Phillips , S. J. Bucci , F. C. Meinzer , and G. Goldstein . 2011. “Hydraulic Capacitance: Biophysics and Functional Significance of Internal Water Sources in Relation to Tree Size.” In Size‐and Age‐Related Changes in Tree Structure and Function, edited by F. C. Meinzer , B. Lachenbruch , and T. E. Dawson , 341–361. Springer.

[pce70647-bib-0083] Scoffoni, C. , C. Albuquerque , C. R. Brodersen , et al. 2017a. “Leaf Vein Xylem Conduit Diameter Influences Susceptibility to Embolism and Hydraulic Decline.” New Phytologist 213, no. 3: 1076–1092. 10.1111/nph.14256 27861926

[pce70647-bib-0084] Scoffoni, C. , C. Albuquerque , C. R. Brodersen , et al. 2017b. “Outside‐Xylem Vulnerability, Not Xylem Embolism, Controls Leaf Hydraulic Decline During Dehydration.” Plant Physiology 173, no. 2: 1197–1210. 10.1104/pp.16.01643 PMC5291720 28049739

[pce70647-bib-0113] Sevanto, S. , N. G. Mcdowell , L. T. Dickman , R. Pangle , and W. T. Pockman . 2014. “How do Trees die? A Test of the Hydraulic Failure and Carbon Starvation Hypotheses.” Plant, cell & environment 37, no. 1: 153–161. 10.1111/pce.12141 PMC4280888 23730972

[pce70647-bib-0085] Siddiq, Z. , Y. J. Zhang , S. D. Zhu , and K. F. Cao . 2019. “Canopy Water Status and Photosynthesis of Tropical Trees Are Associated With Trunk Sapwood Hydraulic Properties.” Plant Physiology and Biochemistry 139: 724–730. 10.1016/j.plaphy.2019.04.031 31055133

[pce70647-bib-0086] Silva, L. M. , L. Pereira , L. Kaack , et al. 2024. “The Potential Link Between Gas Diffusion and Embolism Spread in Angiosperm Xylem: Evidence From Flow‐Centrifuge Experiments and Modelling.” Plant, Cell & Environment 47, no. 12: 4977–4991. 10.1111/pce.15084 39119783

[pce70647-bib-0087] Skelton, R. P. , T. J. Brodribb , and B. Choat . 2017. “Casting Light on Xylem Vulnerability in an Herbaceous Species Reveals a Lack of Segmentation.” New Phytologist 214, no. 2: 561–569. 10.1111/nph.14450 28124474

[pce70647-bib-0088] Sobrado, M. A. 1997. “Embolism Vulnerability in Drought‐Deciduous and Evergreen Species of a Tropical Dry Forest.” Acta Oecologica 18, no. 4: 383–391.

[pce70647-bib-0089] Sperry, J. S. , F. C. Meinzer , and K. A. McCulloh . 2008. “Safety and Efficiency Conflicts in Hydraulic Architecture: Scaling From Tissues to Trees.” Plant, Cell & Environment 31, no. 5: 632–645. 10.1111/j.1365-3040.2007.01765.x 18088335

[pce70647-bib-0090] Sperry, J. S. , and M. T. Tyree . 1988. “Mechanism of Water Stress‐Induced Xylem Embolism.” Plant Physiology 88, no. 3: 581–587. 10.1104/pp.88.3.581 PMC1055628 16666352

[pce70647-bib-0091] Tan, F. S. , H. Q. Song , P. L. Fu , et al. 2020. “Hydraulic Safety Margins of Co‐Occurring Woody Plants in a Tropical Karst Forest Experiencing Frequent Extreme Droughts.” Agricultural and Forest Meteorology 292–293: 108107.

[pce70647-bib-0092] Tavares, J. V. , R. S. Oliveira , M. Mencuccini , et al. 2023. “Basin‐Wide Variation in Tree Hydraulic Safety Margins Predicts the Carbon Balance of Amazon Forests.” Nature 617, no. 7959: 111–117. 10.1038/s41586-023-05971-3 PMC10156596 37100901

[pce70647-bib-0093] Trenberth, K. E. , A. Dai , G. Van Der Schrier , et al. 2014. “Global Warming and Changes in Drought.” Nature Climate Change 4, no. 1: 17–22.

[pce70647-bib-0094] Trifilò, P. , N. Kiorapostolou , F. Petruzzellis , et al. 2019. “Hydraulic Recovery From Xylem Embolism in Excised Branches of Twelve Woody Species: Relationships With Parenchyma Cells and Non‐Structural Carbohydrates.” Plant Physiology and Biochemistry 139: 513–520. 10.1016/j.plaphy.2019.04.013 31015090

[pce70647-bib-0096] Tyree, M. T. , and J. S. Sperry . 1988. “Do Woody Plants Operate Near the Point of Catastrophic Xylem Dysfunction Caused by Dynamic Water Stress? Answers From a Model.” Plant Physiology 88, no. 3: 574–580. 10.1104/pp.88.3.574 PMC1055627 16666351

[pce70647-bib-0097] Tyree, M. T. , and J. S. Sperry . 1989. “Vulnerability of Xylem to Cavitation and Embolism.” Annual Review of Plant Physiology and Plant Molecular Biology 40, no. 1: 19–36.

[pce70647-bib-0098] Tyree, M. T. , and S. Yang . 1990. “Water‐Storage Capacity of Thuja, Tsuga and Acer Stems Measured by Dehydration Isotherms: The Contribution of Capillary Water and Cavitation.” Planta 182: 420–426. 10.1007/BF02411394 24197194

[pce70647-bib-0099] Tyree, M. T. , and M. H. Zimmermann . 2002. “Hydraulic Architecture of Whole Plants and Plant Performance.” In Xylem Structure and the Ascent of Sap 2nd ed, 175–214. Springer.

[pce70647-bib-0100] Tyree, M. T. , and M. H. Zimmermann . 2013. Xylem Structure and the Ascent of Sap. Springer Science & Business Media.

[pce70647-bib-0101] Urli, M. , A. J. Porte , H. Cochard , Y. Guengant , R. Burlett , and S. Delzon . 2013. “Xylem Embolism Threshold for Catastrophic Hydraulic Failure in Angiosperm Trees.” Tree Physiology 33, no. 7: 672–683. 10.1093/treephys/tpt030 23658197

[pce70647-bib-0102] Vergeynst, L. L. , M. Dierick , J. A. N. Bogaerts , V. Cnudde , and K. Steppe . 2015. “Cavitation: A Blessing in Disguise? New Method to Establish Vulnerability Curves and Assess Hydraulic Capacitance of Woody Tissues.” Tree Physiology 35, no. 4: 400–409. 10.1093/treephys/tpu056 25030935

[pce70647-bib-0103] Wang, Y. , J. Liu , and M. T. Tyree . 2015. “Stem Hydraulic Conductivity Depends on the Pressure at Which It Is Measured and How This Dependence Can Be Used to Assess the Tempo of Bubble Pressurization in Recently Cavitated Vessels.” Plant Physiology 169, no. 4: 2597–2607. 10.1104/pp.15.00875 PMC4677890 26468516

[pce70647-bib-0104] Wang, Y. , R. Pan , and M. T. Tyree . 2015. “Studies on the Tempo of Bubble Formation in Recently Cavitated Vessels: A Model to Predict the Pressure of Air Bubbles.” Plant Physiology 168, no. 2: 521–531. 10.1104/pp.114.256602 PMC4453774 25907963

[pce70647-bib-0105] Wei, Y. , Y. J. Chen , Z. Siddiq , et al. 2023. “Hydraulic Traits and Photosynthesis Are Coordinated With Trunk Sapwood Capacitance in Tropical Tree Species.” Tree Physiology 43, no. 12: 2109–2120. 10.1093/treephys/tpad107 37672225

[pce70647-bib-0106] WFO . 2023. World Flora Online. Version v.2023.12 (December 22, 2023). http://www.worldfloraonline.org.

[pce70647-bib-0107] Williams, C. B. , R. Reese Næsborg , and T. E. Dawson . 2017. “Coping With Gravity: The Foliar Water Relations of Giant Sequoia.” Tree Physiology 37, no. 10: 1312–1326. 10.1093/treephys/tpx074 28985377

[pce70647-bib-0108] Yao, G. Q. , Z. F. Nie , N. C. Turner , et al. 2021. “Combined High Leaf Hydraulic Safety and Efficiency Provides Drought Tolerance in Caragana Species Adapted to Low Mean Annual Precipitation.” New Phytologist 229, no. 1: 230–244. 10.1111/nph.16845 PMC7754512 32749703

[pce70647-bib-0109] Ziegler, C. , S. Coste , C. Stahl , et al. 2019. “Large Hydraulic Safety Margins Protect Neotropical Canopy Rainforest Tree Species Against Hydraulic Failure During Drought.” Annals of Forest Science 76: 115.

[pce70647-bib-0110] Ziegler, C. , S. Levionnois , D. Bonal , P. Heuret , C. Stahl , and S. Coste . 2023. “Large Leaf Hydraulic Safety Margins Limit the Risk of Drought‐Induced Leaf Hydraulic Dysfunction in Neotropical Rainforest Canopy Tree Species.” Functional Ecology 37, no. 6: 1717–1731.

[pce70647-bib-0111] Ziemińska, K. , M. Westoby , and I. J. Wright . 2015. “Broad Anatomical Variation Within a Narrow Wood Density Range—A Study of Twig Wood Across 69 Australian Angiosperms.” PLoS One 10, no. 4: e0124892. 10.1371/journal.pone.0124892 PMC4408027 25906320

[pce70647-bib-0112] Zimmermann, M. H. 1983. Xylem Structure and the Ascent of Sap. Springer‐Verlag, New York.

