# Supplementary material for: Water Released From Vessel Embolism May Temporarily Halt Further Embolism in Tropical Dipterocarp Trees
Source: Plant Cell Environ. 2026 Jul 2;49(11):7855–70. doi: 10.1111/pce.70647 (PMC13646656; doi:10.1111/pce.70647)
Supplement: Supplementary file 1 [file PCE-49-7855-s001.docx]

**Supporting Information**

**Figure S1:** Tree height (m) and branch sampling locations in each of the sampled trees. Each bar represents one tree. Red = *Dipterocarpus caudiferus*, yellow = *Dryobalanops lanceolata*, green = *Parashorea malaanonan*, blue = *Parashorea tomentella*, purple = *Rubroshorea johorensis*. Letters to the right of each bar indicate branch sampling height (m) and location (i.e., top (T), middle (M) or bottom (B) of the tree canopy).

**Figure S2:** Changes in ψ_branch_, temperature and relative humidity over time for the subpanels in Figure 1_a_ (a) and 1_b_ (b).

**Figure S3:** (a) shows the increases in ψ_branch_ (Δψ_branch_ >0 MPa, measured every 10 minutes) plotted against extrapolated changes in temperature (measured every 5 minutes). Pearson's correlation analysis indicated no significant correlation (r = -0.000, t = -0.001, df = 9747, p = 1.00). (b) shows the same figure as (a) but focuses only on the larger increases (Δψ_branch_ >0.1 MPa). Pearson's correlation analysis indicated no significant correlation (r = -0.013, t = -0.310, df = 585, p = 0.76). (c) shows the increases in ψ_branch_ (Δψ_branch_ >0 MPa, measured every 10 minutes) plotted against extrapolated changes in relative humidity (measured every 5 minutes). Pearson's correlation analysis indicated no significant correlation (r = 0.003, t = 0.324, df = 9747, p = 0.75). (d) shows the same figure as (c) but focuses only on the larger increases (Δψ_branch_ >0.1 MPa). Pearson's correlation analysis indicated no significant correlation (r = 0.008, t = 0.185, df = 585, p = 0.85).

**Figure S4:** Changes in ψ_branch_ over time, measured every 10 minutes, for five additional example curves (a-e). Points in time where ψ_branch_ increased are coloured in red. The corresponding leaf vulnerability curves (ψ_branch_ plotted against cumulative % leaf embolism) for the examples in (a-e) are also given (f-j). Points in the vulnerability curve where ψ_branch_ increased are coloured in red.

**Figure S5:** Changes in ψ_branch_ over time (positive Δψ_branch_, MPa), measured every 10 min, plotted against ψ_branch_ (MPa) for all curves within each respective species (a-e). The colour of points represents the density of observations. The vertical lines represent the mean hydraulic thresholds within each species, where blue = TLP; red solid = branch P_50_; red dashed = branch P_12_ and P_88_; green solid = leaf P_50_; green dashed = leaf P_12_ and P_88_. Panels (f-j) show, for all curves within each respective species, changes in ψ_branch_ over time, measured every 10 min, plotted against the difference between leaf P_50_ and the ψ_branch_ at which the respective increase in ψ_branch_ occurred, with positive values along the x-axis indicating that the respective increase in ψ_branch_ occurred before leaf P_50_ (this difference was evaluated within each respective curve using the curve-specific leaf P_50_). Dashed lines represent a ±0.1 MPa interval around leaf P_50_.

**Figure S6:** The water content (mean ±SD) per cm of branch length, termed ‘standardised water content’ and derived from measurements of saturated water content for the sapwood and bark at 1 m distance from the tip of branches, is shown for each species as a stacked bar graph (a). Red = Dipterocarpus caudiferus, yellow = Dryobalanops lanceolata, green = Parashorea malaanonan, blue = Parashorea tomentella, purple = Rubroshorea johorensis. The top and bottom part of each bar represents the contribution of bark (B) and sapwood (S), respectively (mean ±SD). The contribution of vessels (V) specifically (mean ±SD), which are included in the sapwood, is plotted within the sapwood portion for each bar. The relative contribution (mean ±SD) of these tissues to standardised water content is shown as a fraction (unitless) in (b).

**Table S1:** Paired comparisons of leaf and branch P_50_ values for the 69 of 78 samples for which paired traits data were available. Mean ± SD P_50_ values (MPa) for leaves and branches are reported along with the Wilcoxon signed-rank test statistic (V), the mean paired difference (MPa) with its 95% confidence intervals (where negative values indicate that leaf P_50_ is more negative than branch P_50_), sample size (n), and associated p-value. Significant p-values are shown in bold.

| **Species** | **Leaf *P*_50_ (± SD)** | **Branch *P*_50_ (± SD)** | **Test Statistic** | **Mean Difference (± 95% CI)** | **n** | **p** |
| --- | --- | --- | --- | --- | --- | --- |
| All | -2.97 (± 0.40) | -2.71 (± 0.50) | V = 469 | -0.26 (± 0.13) | 69 | **p < 0.001** |
| *Dipterocarpus caudiferus* | -2.72 (± 0.10) | -2.58 (± 0.44) | V = 11 | -0.14 (± 0.32) | 8 | p = 0.383 |
| *Dryobalanops lanceolata* | -3.22 (± 0.56) | -2.58 (± 0.47) | V = 0 | -0.64 (± 0.18) | 12 | **p < 0.001** |
| *Parashorea malaanonan* | -3.25 (± 0.27) | -2.82 (± 0.42) | V = 6 | -0.43 (± 0.31) | 11 | **p < 0.05** |
| *Parashorea tomentella* | -2.94 (± 0.24) | -2.69 (± 0.55) | V = 34 | -0.24 (± 0.22) | 19 | **p < 0.05** |
| *Rubroshorea johorensis* | -2.78 (± 0.39) | -2.80 (± 0.56) | V = 80 | 0.01 (± 0.28) | 19 | p = 0.568 |

**Table S2:** Model parameters, including their unit, equation, overall and intraspecific mean (± 95% confidence intervals).

| **Parameter** | **Mean** | **Equation** |
| --- | --- | --- |
| **Xylem diameter ( X_dia_ )** | **m** |  |
| Overall | 9.56e-03 ± 1.12e-03 |  |
| *Dipterocarpus caudiferus* | 1.11e-02 ± 3.19e-03 |  |
| *Dryobalanops lanceolata* | 7.99e-03 ± 1.45e-03 |  |
| *Parashorea malaanonan* | 6.90e-03 ± 1.32e-03 |  |
| *Parashorea tomentella* | 1.19e-02 ± 3.44e-03 |  |
| *Rubroshorea johorensis* | 8.95e-03 ± 1.25e-03 |  |
| **Xylem area ( X_a_ )** | **m^2^** | **π * (X_dia_ / 2)^2^** |
| Overall | 9.00e-05 ± 3.15e-05 |  |
| *Dipterocarpus caudiferus* | 1.12e-04 ± 6.64e-05 |  |
| *Dryobalanops lanceolata* | 5.47e-05 ± 1.66e-05 |  |
| *Parashorea malaanonan* | 4.02e-05 ± 1.47e-05 |  |
| *Parashorea tomentella* | 1.51e-04 ± 1.13e-04 |  |
| *Rubroshorea johorensis* | 6.80e-05 ± 1.80e-05 |  |
| **Vessel lumen fraction ( VLF )** | **fraction** |  |
| Overall | 1.63e-01 ± 7.61e-03 |  |
| *Dipterocarpus caudiferus* | 1.41e-01 ± 1.52e-02 |  |
| *Dryobalanops lanceolata* | 1.92e-01 ± 1.84e-02 |  |
| *Parashorea malaanonan* | 1.60e-01 ± 1.57e-02 |  |
| *Parashorea tomentella* | 1.70e-01 ± 1.50e-02 |  |
| *Rubroshorea johorensis* | 1.51e-01 ± 1.49e-02 |  |
| **Xylem lumen volume ( X_lv_ )** | **m^3^** | **VLF * X_a_ * segment length** |
| Overall | 1.55e-05 ± 6.82e-06 |  |
| *Dipterocarpus caudiferus* | 1.65e-05 ± 1.07e-05 |  |
| *Dryobalanops lanceolata* | 1.11e-05 ± 3.39e-06 |  |
| *Parashorea malaanonan* | 6.34e-06 ± 2.37e-06 |  |
| *Parashorea tomentella* | 2.82e-05 ± 2.48e-05 |  |
| *Rubroshorea johorensis* | 9.83e-06 ± 2.36e-06 |  |
| **Total branch water ( TBW )** | **g** | **X_lv_ * 1000 * 1000** |
| Overall | 12.55 ± 2.32 |  |
| *Dipterocarpus caudiferus* | 16.62 ± 10.63 |  |
| *Dryobalanops lanceolata* | 11.44 ± 3.52 |  |
| *Parashorea malaanonan* | 6.56 ± 2.45 |  |
| *Parashorea tomentella* | 16.95 ± 6.11 |  |
| *Rubroshorea johorensis* | 10.22 ± 2.47 |  |
| **Total leaf area ( LA )** | **m^2^** |  |
| Overall | 0.57 ± 0.08 |  |
| *Dipterocarpus caudiferus* | 0.55 ± 0.23 |  |
| *Dryobalanops lanceolata* | 0.59 ± 0.23 |  |
| *Parashorea malaanonan* | 0.36 ± 0.14 |  |
| *Parashorea tomentella* | 0.72 ± 0.22 |  |
| *Rubroshorea johorensis* | 0.56 ± 0.15 |  |
| **Leaf capacitance ( C_leaf_ )** | **g g^-1^ MPa^-1^** |  |
| Overall | 0.09 ± 0.01 |  |
| *Dipterocarpus caudiferus* | 0.07 ± 0.02 |  |
| *Dryobalanops lanceolata* | 0.08 ± 0.03 |  |
| *Parashorea malaanonan* | 0.08 ± 0.02 |  |
| *Parashorea tomentella* | 0.08 ± 0.02 |  |
| *Rubroshorea johorensis* | 0.13 ± 0.04 |  |
| **Leaf mass per area ( LMA )** | **g m^-2^** |  |
| Overall | 118.82 ± 8.85 |  |
| *Dipterocarpus caudiferus* | 101.69 ± 14.86 |  |
| *Dryobalanops lanceolata* | 109.02 ± 12.34 |  |
| *Parashorea malaanonan* | 105.51 ± 27.58 |  |
| *Parashorea tomentella* | 149.29 ± 26.96 |  |
| *Rubroshorea johorensis* | 116.91 ± 7.35 |  |
| **Area based leaf capacitance ( C_area_ )** | **g m^-2^ MPa^-1^** | **C_leaf_ * LMA** |
| Overall | 11.22 ± 2.58 |  |
| *Dipterocarpus caudiferus* | 6.46 ± 1.58 |  |
| *Dryobalanops lanceolata* | 9.61 ± 4.27 |  |
| *Parashorea malaanonan* | 7.60 ± 2.03 |  |
| *Parashorea tomentella* | 14.11 ± 8.90 |  |
| *Rubroshorea johorensis* | 14.87 ± 5.28 |  |
| **Total leaf capacitance ( C_branch_ )** | **g MPa^-1^** | **C_area_ * LA** |
| Overall | 5.95 ± 1.33 |  |
| *Dipterocarpus caudiferus* | 3.72 ± 1.94 |  |
| *Dryobalanops lanceolata* | 4.96 ± 2.02 |  |
| *Parashorea malaanonan* | 2.56 ± 1.16 |  |
| *Parashorea tomentella* | 7.78 ± 2.52 |  |
| *Rubroshorea johorensis* | 8.49 ± 4.16 |  |
| **Leaf water potential increase** | **MPa** | **TBW * % vessel embolism / C_branch_** |

**Table S3:** Results of the analysis of the branch height effect on the mean and cumulative increase in ψ_branch_ for Dipterocarpus caudiferus (the only species that showed significant relationships). Results are from linear regression models with branch height as fixed effect. R^2m^ and R^2a^ represent the multiple R^2^ and adjusted R^2^, respectively. Values for the fixed term intercept and slope are presented as estimate ± standard error.

| **Mean increase in ψ_branch_** | | | | | | |
| --- | --- | --- | --- | --- | --- | --- |
| **Species** | **Intercept** | **Slope for branch height** | **R^2m^** | **R^2a^** | **p** | **df** |
| *Dipterocarpus caudiferus* | 0.013 ± 0.005 | 0.001 ±0.000 | 0.67 | 0.63 | **0.002** | 9 |
| **Cumulative increase in ψ_branch_** | | | | | | |
| **Species** | **Intercept** | **Slope for branch height** | **R^2m^** | **R^2a^** | **p** | **df** |
| *Dipterocarpus caudiferus* | 1.767 ± 1.133 | 0.069 ±0.026 | 0.45 | 0.39 | **0.024** | 9 |
